# Sustained Effector Functions and Memory Accumulation of αβ T Cells in Children With Congenital Heart Disease

**DOI:** 10.1002/eji.70209

**Published:** 2026-05-13

**Authors:** Yusuf Eshimutu Abu, Christoph Kammeyer, Tao Yang, Rodrigo Gutierrez Jauregui, Alexa Cramer, Claudia Junge, Svea Kleiner, Anika Janssen, Adan Chari Jirmo, Alexander Horke, Reinhold Förster, Philipp Beerbaum, Martin Boehne, Sarina Ravens

**Affiliations:** ^1^ Institute of Immunology Hannover Medical School Hannover Germany; ^2^ Department of Pediatric Cardiology and Intensive Care Medicine Hannover Medical School Hannover Germany; ^3^ Department of Pediatric Surgery Hannover Medical School Hannover Germany; ^4^ Department of Cardiothoracic Transplantation and Vascular Surgery Hannover Medical School Hannover Germany; ^5^ Cluster of Excellence RESIST (EXC 2155) Hannover Medical School Hannover Germany

**Keywords:** congenital heart disease, T cells, thymectomy, immune cell profiling

## Abstract

Congenital heart disease (CHD) is a major global health problem. Although treatment and survival have impressively improved, many patients face comorbidities such as increased susceptibility to infection that may shorten their lives. As many children undergo cardiac surgery with concomitant thymectomy early in life, most studies of the immune system in patients with CHD have focused on the quantitative analysis of lymphocyte subpopulations, maturation, and T cell receptor repertoires, whereas knowledge of effector functions remains limited. We analysed αβ T cell phenotypes, transcriptomes, and functions in children with CHD who underwent cardiac surgery within a year of birth and were followed up to five to ten years after thymectomy, in comparison to age‐matched healthy controls.

Children with CHD showed reduced T cell populations, a reduction of recent thymic emigrants (RTEs) and naive T cells, regulatory T cells with a higher suppressive phenotype, and, most importantly, high activation states of T cells, further reflected in higher granzyme and cytokine production. This reveals persistent alterations in T cell immunity years after early‐life thymectomy in children with CHD, highlighting the need for long‐term immune monitoring and providing a basis for understanding immune‐related comorbidities in this patient population.

## Graphical abstract

This study showed dysregulated αβ T cell homeostasis with ongoing T cell activation and low‐grade inflammation in 5‐12‐year‐old CHD children who underwent early‐life cardiac surgery with thymectomy, characterized by reduced naïve T cells and increased memory/effector and senescent‐like T cells. There was also higher expression of inhibitory receptors on effector T cells and elevated suppressive gene expression in Tregs that may regulate immune overactivation.

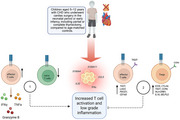

## 1 Introduction

Congenital heart disease (CHD) is the most prevalent congenital anomaly, affecting approximately one out of every 100 newborns [1]. It includes a wide range of developmental defects of the heart and great vessels. For the majority of children, particularly those with severe and complex CHD, early‐life cardiac surgery is necessary for both corrective and palliative purposes [2]. During congenital heart surgery, the removal of thymic tissue—either completely or partially is usually required to ensure adequate surgical access to the heart and great vessels.

The thymus is the principal organ in which common T cell progenitors give rise to T cells that express T cell receptors (TCRs) [3], the majority of which express a TCR composed of an α‐ and β‐chain. The development of αβ T cells results in either CD4 or CD8 T cells. Both subsets predominantly leave the thymus as naïve T cells and undergo peripheral maturation into effector T cells upon antigen‐mediated activation of their TCR [4]. The activity of the thymus decreases with age as its tissue mass, cellularity, and organization all decrease, which corresponds to a reduction in the T cell output [5, 6]. The declining thymic output during aging leads to contraction of the naïve T cells, and the maintenance of the naïve T cell pool relies on peripheral division of existing clones rather than on de novo production of new ones [7]. Homeostatic proliferation mediates the maintenance of T cell numbers in the periphery to compensate for a lymphopenic condition [8]. This thymic involution is one of the identifiable features of the aging T cell compartment and immune system. It might be associated with an increased susceptibility to infection, autoimmune disease, and cancer [9].

Questions regarding the immune status and long‐term consequences of children with CHD who underwent congenital heart surgery, in which partial or complete removal of the thymus (thymectomy) was indicated, have recently attracted interest, given that 97% of children with CHD now survive into adulthood [10, 11]. Recent population studies suggest an association between CHD and potential thymectomy and an elevated risk of all‐cause mortality, autoimmune diseases, infections, and cancer later in life, attributable to an altered immune competence [10, 12, 13]. This is most likely related to the perturbation of T cell development postsurgery, and the inflammatory state in these children [14, 15]. Another recently published study reported higher hospitalisation rates due to infections, pneumonia, wheezing, and asthma in children after a potential thymectomy than population‐based controls [16]. While children with CHD who have undergone thymectomy showed an adequate T cell response to influenza vaccination later in life [17], other studies demonstrated a diminished response to vaccination against tick‐borne encephalitis [18].

Several studies have attempted to determine the impact of surgical procedures affecting the thymus in patients with CHD on T‐cell phenotypes and responsiveness [10]. Halnon et al. [19] indicate that long‐term maintenance of lymphocyte populations is disrupted by congenital heart surgery, particularly if involving thymectomy. Other studies have also described changes to the T cell phenotype, characterized by a decline in naïve T cells and an increase in memory T cells, along with reduced immune repertoire diversity, in patients with CHD who underwent a (partial) thymectomy as neonates or infants [18, 20, 21, 22, 23]. Inadequate production of naïve immune cells in the thymus results in expansion of memory T cells and an accumulation of antigen exposure on the remaining T cells, which may, in turn, enhance immune senescence [5, 24, 25]. Altogether, characterization of immunity in individuals with CHD has mainly focused on the quantitative analysis of lymphocyte subpopulations, maturation, and T cell receptor repertoire [10], whereas knowledge on effector T‐cell functionality remains limited. In this study, we aimed to fill this gap by investigating the transcriptomes, surface phenotypes, and cytokine production capacity of αβ T cells in children with CHD.

## 2 Materials and Methods

### 2.1 The Study Cohorts

The study included a mix of male and female children with congenital heart disease, and age‐matched control was assessed. Sex as a biological variable was not considered in this study. The study focused on immunological changes in school‐age children with congenital heart disease (CHD) who underwent cardiac surgery in the neonatal period or early infancy with concomitant partial or complete thymectomy. School‐age children with CHD between the ages of 5 and 12 who visited the pediatric cardiology outpatient clinic for a routine follow‐up appointment were eligible for inclusion (*n* = 43). The children were diagnosed with a wide spectrum of CHD, such as simple transposition of great arteries (TGA), complex TGA, double outlet right ventricle (DORV), tetralogy of Fallot (TOF), or ventricular septal defect (VSD). During congenital heart surgery, all children underwent thymectomy to facilitate surgical access. However, the extent of the thymectomy was not documented in detail in the medical records. Age‐matched (*n* = 17), generally healthy children who had a blood sample taken before planned minor surgery at the Department of Pediatric Surgery, Hannover Medical School, Hannover, Germany, were included in the study as non‐CHD controls (Ctrl). Children with genetic disorders, suspected or diagnosed immunodeficiencies, acute or chronic infections, or a history of prematurity were excluded from the study. Clinical data were drawn from a standardized questionnaire completed by parents during the study visit and from patients’ health records.

### 2.2 Isolation of Plasma

Plasma samples were obtained from freshly collected blood samples. Plasma was separated from whole blood by centrifugation at 3000 *g* for 5 min. The supernatant was aliquoted and stored at –80°C until analysis.

Isolation of peripheral mononuclear blood cells: Peripheral blood mononuclear cells (PBMCs) were freshly isolated from EDTA blood via density medium centrifugation. Isolated cells were gently frozen in a freezing medium containing 90% heat‐inactivated fetal calf serum (FCS) and 10% dimethyl sulfoxide (DMSO), and stored in aliquots at −80°C until further processing.

### 2.3 Multicolor Flow Cytometry

PBMCs were thawed in a 37°C water bath, prepared by washing in 0.3% FCS (Sigma), 3 mM EDTA (Carl Roth GmbH + Co. KG), PBS buffer, and apportioned in 1 × 10^6^ cells per participant for subsequent spectral flow cytometric analysis, and in >1 × 10^6^ cells for sorting of αβ and γδ T cell populations. After thawing, samples were stained for 20 min at 4°C in the refrigerator with an antibody mix containing 26 and 17 monoclonal antibodies and a viability dye for cell phenotypic characterization and stimulation assay, respectively (Tables S1 and S2). For intracellular staining, the cells were surface‐stained, fixed, and permeabilized with eBioscience Intracellular Fixation & Permeabilization Buffer Set (eBioscience) according to the manufacturer's instructions. The cells were stained with the intracellular antibodies for 30 min at 4°C in a refrigerator (Table S3). Acquisition, unmixing, and compensating were performed on the Cytek Aurora spectral flow cytometer using Spectroflow software (Cytek Biosciences). Subsequent gating of cell populations of interest was performed using FlowJo software (version 10.10).

For isolation of the described populations on an FACS Aria Fusion (BD), thawed PBMCs were stained with antibodies directed against the following surface antibodies: CD3 (BV510, UCHT1, BioLegend, 1:75), TCRγδ (PE, REA591, Miltenyi, 1:75), TCRαβ (FITC, WT31, BD, 1:75), CD19 (PE‐Cy7, HIB19, eBioscience, 1:75); dead cells were detected via staining with Zombie DAPI.

### 2.4 In Vitro Stimulation and Functional Assays

For stimulation experiments, PBMCs were thawed, washed, and resuspended in advanced RPMI medium (RPMI 1640, Gibco), supplemented with 10% heat‐inactivated FCS (Sigma), 1% Penicillin‐Streptomycin (Gibco), 1% GlutaMAX (Gibco), and 3.5 × 10^−6^ % β‐Mercaptoethanol (Sigma). For Phorbol myristate acetate (PMA) and Ionomycin Stimulation, cells were cultured at 5 × 10^5^ cells per well in a 96‐well U‐bottom plate. In one well, 250 µL of RPMI‐1640 containing PMA at a concentration of 50 ng/mL, Ionomycin at a concentration of 1 µg/mL, and Brefeldin A (BFA) at 10 µg was stimulated at 37°C for 3 h, and the second well was without the stimulant. For anti‐CD3 and anti‐CD28 stimulation, anti‐CD3 (Biolegend 300438, clone UCHT1, concentration = 2.94 mg/mL) and anti‐CD28 (Biolegend 302934, clone CD28.2, Concentration = 2.82 mg/mL) were diluted in sterile PBS to obtain a coating concentration of 5.88 and 2.82 µg/mL, respectively. A 48‐well plate was coated overnight at 4°C and washed to remove excess antibodies. Total PBMC was resuspended in the medium containing 10 µg/mL BFA, and 1 × 10^6^ cells/mL was seeded in the respective coated wells and incubated for 6 h at 37°C. Expressed surface molecules, intracellular cytokines, and frequencies of CD4 and CD8 T cells were determined using flow cytometry. Acquisition, unmixing, and compensating were performed on the Cytek Aurora spectral flow cytometer, as described for the phenotyping experiment.

### 2.5 LEGENDplex Assay to Measure Inflammatory Cytokines

To quantify cytokines secreted in plasma, we used LEGENDplex (BioLegend, 740809), which is a flow cytometry‐based 13‐plex multianalyte kit able to detect IL‐1β, IFN‐α2, IFNγ, TNFα, IL‐6, IL‐18, IL‐10, IL‐17A, IL‐12p70, IL‐23, IL‐33, CCL2, and CXCL8. Equal volumes of assay buffer, plasma, and capture beads were loaded onto wells of a 96‐well V‐bottom plate and incubated at room temperature on a plate shaker set to 650 rpm. Afterwards, plates were centrifuged at 300 relative centrifugal force (RCF) and washed with assay buffer. Wells were incubated for 1 h at room temperature with detection antibodies, followed by the addition of biotinylated detection antibody. After an additional 30 min of incubation, plates were centrifuged at 250 RCF and washed with assay buffer. Wells were resuspended in wash buffer before flow cytometry analysis using a Cytek Aurora spectral flow cytometer (Cytek Biosciences). The machine was set up according to the kit manufacturer's instructions using setup beads. Values for each cytokine were obtained using the LEGENDplex data analysis software (biolegend.com/en‐us/Legendplex). All samples were performed in duplicate. The detection limit of each cytokine is shown in Table S4.

### 2.6 scRNAseq Library Generation and Data Analysis

Peripheral blood mononuclear cells (PBMCs) were obtained from three children with CHD who underwent cardiac surgery with thymectomy within one year of birth. The PBMCs were thawed, and fluorescence‐activated cell sorting (FACS) was performed to isolate live αβ and γδ T cells. These sorted T cells were then used to generate single‐cell RNA sequencing (scRNAseq) libraries using Chromium Next GEM Single Cell V(D)J Reagent Kits v2, following the manufacturer's protocol (10× Genomics) for the study performed here. The scRNAseq libraries were sequenced on the NovaSeq X platform, targeting 25,000 read pairs per cell. Reads were aligned to the reference genome GRCh38, and the gene expression matrix was generated using cellranger‐8.0.1 (10× Genomics). Subsequent analysis was performed on αβ T cells using Seurat v5.0.16 [26, 27] in R 4.3.2. For comparative analysis, an age‐matched non‐CHD public αβ T cell dataset (GSM8443049) was used to compare with CHD patients. Batch effects were corrected using the R package Harmony [28], using the default parameters and group.by.vars = “dataset”, where “dataset” corresponds to the batch. Low‐quality cells were identified and excluded based on mitochondrial gene content over 10%, or fewer than 200, or more than 4000 detected genes. DoubletFinder was used to identify doublets using the default parameters, which were removed to improve differential gene expression analysis performance [29]. To remove other contamination from B cells or myeloid cells, the data were further filtered out. Datasets were merged, normalized, and scaled using linear transformation using ScaleData, followed by principal component analysis (PCA) and dimension reduction using RunPCA and RunUMAP (resolution = 0.8) functions, respectively.

### 2.7 Subclustering

A TCR expression gene module score was used to isolate αβ T cells from total T cells by excluding γδ T cells, as described by Song et al. [30], and subset function was used to isolate αβ T cells. A standard Seurat workflow was used to recluster the αβ T cell for downstream analysis. Annotation of the αβ T cell clusters was based on the gene expression profiles of lineage markers. Differentially expressed gene analysis was performed with the Seurat function FindMarkers/FindAllMarkers. Genes expressed in at least 25% of the clusters and having an adjusted *p*‐value <0.05 (Benjamini–Hochberg correction) were considered significant. Similar clusters were merged for onward characterization.

### 2.8 Differential Gene Expression Analysis Between Age Groups

For transcriptional changes between CHD and Ctrl, the subset function in the Seurat package was used to obtain the respective clusters, and differentially expressed genes (DEGs) were identified using Seurat's FindMarkers function (min.pct = 0.25, logfc.threshold = 0.25, test.use = MAST). Genes were assigned to CHD or Ctrl based on the sign of avg_log2FC. Module scores for selected gene sets were computed using Seurat's AddModuleScore function.

### 2.9 Gene Ontology (GO) Enrichment Analysis

Functional enrichment of DEGs was performed using the compareCluster function in the clusterProfiler [31] R package (enrichGO, ontology = Biological Process, OrgDb = *org.Hs.eg.db*, keyType = “SYMBOL”, pAdjustMethod = “BH”, qvalueCutoff = 0.05). Gene ratios were calculated as the percentage of DEGs annotated to each term relative to the total background genes. Selected Gene Ontology (GO) terms of interest were visualized using ggplot2.

### 2.10 Statistics Analysis

Patient demographics and clinical data were summarized using descriptive statistics. Group comparisons of cell frequencies and counts between CHD and control participants were performed using the Mann–Whitney test due to data distribution and sample size. In all statistical tests, a *p*‐value of <0.05 was considered statistically significant. Analysis of the difference between the proportion of cells in clusters between Ctrl and CHD scRNA‐seq data was done using the scProportion Test [32]. For each cluster, statistical significance was assessed using a permutation test, while the confidence interval for the magnitude difference was obtained through bootstrapping. All analyses were conducted with the statistical software R (version 4.0.3).

### 2.11 Study Approval

This prospective observational study was conducted at the Department of Pediatric Cardiology and Intensive Care Medicine and the Institute of Immunology, Hannover Medical School, Hannover, Germany. The research protocol was approved by the institutional ethics board at Hannover Medical School (10198_BO_S_2022). All procedures involving human participants were in accordance with the ethical standards of the institutional and/or national research committee and with the 1964 Helsinki Declaration and its later amendments or comparable ethical standards. Written informed consent was obtained for children from their legal guardians, and, depending on age, the children as well.

## 3 Results

### 3.1 Reduced Thymic Activity and Perturbation of the T Cell Pool in Children With CHD

**FIGURE 1:**
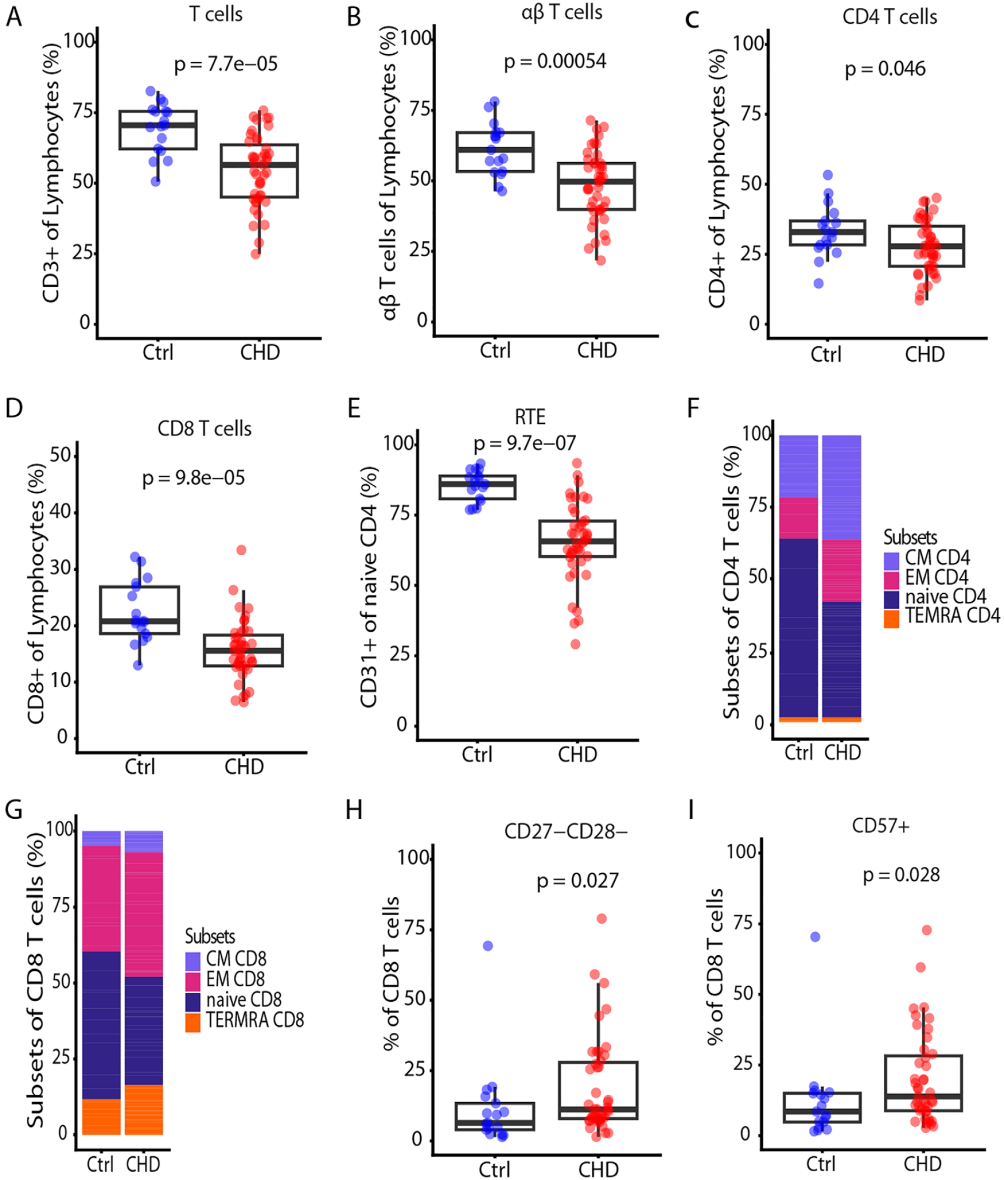
Thymectomy leads to reduced T cell subsets and lower thymic output. Flow cytometry analysis of peripheral mononuclear blood cells from 5–12‐year‐old children with CHD (*n* = 43, CHD), after cardiac surgery involving total or partial thymectomy, and age‐matched non‐CHD controls (*n* = 17, Ctrl). (A) Boxplot showing the percentage of CD3 T cells within live lymphocytes. (B) Boxplot showing the percentage of αβ T cells within live lymphocytes. (C) Percentage of CD4 T cells within live lymphocytes. (D) Percentage of CD8 T cells within live lymphocytes. (E) Percentage of CD31^+^ cells within naive CD4 T cells, indicative of Recent Thymic Emigrants (RTE). (F) Stacked bar plot showing the percentage of CD4 T cells with a naïve (CD45RA^+^CCR7^+^), central memory [CM CD4] (CD45RA^−^ CCR7^+^), effector memory [EM CD4] (CD45RA^−^ CCR7^−^), and terminally differentiated effector memory phenotype [TEMRA CD4] (CD45RA^+^CCR7^−^). (G) Stacked bar plot showing the percentage of CD8 T cells with a naïve (CD45RA^+^CCR7^+^), central memory [CM CD8] (CD45RA^−^ CCR7^+^), effector memory [EM CD8] (CD45RA^−^ CCR7^−^), and terminally differentiated effector memory phenotype [TEMRA CD8] (CD45RA^+^CCR7^−^). (H) Percentage of CD27^−^CD28^−^ CD8 T cells. **(I**) Percentage of CD57^+^ CD8 T cells. Statistical analyses were performed using the Mann–Whitney *U* test; *p*‐value < 0.05 was considered significant. Horizontal bars indicate median values. Each dot represents one donor.

Peripheral blood T cells were studied by flow cytometry in 6‐ to 12‐year‐old children who underwent cardiac surgery for CHD with partial removal of the thymus when aged under 1 year (*n* = 43) and compared with age‐matched controls (*n* = 17) (Table 1). Flow cytometric analysis (Figure S1A) showed a significant reduction of total CD3 T cells, αβ T cells, CD4 T cells, and CD8 T cells among lymphocytes (Figure 1A–D), and no difference in γδ T cells among lymphocytes was observed (Figure S1B). In addition, the measurement of CD31 expression on naive CD4 T cells, which associates with newly generated T cells, depicts a significant reduction of recent thymic emigrants in CHD (Figure 1E). Together, this indicates a reduced thymic activity in these children. Notably, the distribution of T cell subsets itself was not disturbed, as reflected by the relatively similar frequencies of CD4 and CD8 T cells, and γδ T cells among CD3 T cells in the CHD and control group (Figure S1C–E). Furthermore, analysis of a subset of the cohort CHD (*n* = 21) and the controls (*n* = 9) reflects similar cell counts and proportions of the total T cells (Figure S2A), αβ T cells (Figure S2B), CD4 T cells (Figure S2C), and CD8 T cells (Figure S2D).

**TABLE 1:** Summarized characteristics of children with CHD postsurgery and controls used for cell stimulation assays, multicolor flow cytometry, and FACS.

Characteristic	Children with CHD (*n* = 43)	Ctrl (*n* = 17)
Age in years at sample collection: Median (IQR)	7.0 (5.0)	8.0 (6.0)
Age in months at time point of cardiac surgery involving (partial) removal of thymic tissue: Median (IQR)	2.00 (5.00)	NA
Sex (M/F)	28/15	11/6

Abbreviations: CHD: congenital heart disease, Ctrl: age‐matched controls, NA: not applicable, M: male, F: female.

Next, we characterized the T cell phenotypes via CCR7 and CD45RA surface expression used to define naïve and memory T cells [33]. Here, we observed lower frequencies of naïve CD4 and CD8 T cells (CD45RA^+^CCR7^+^) and increased memory T cells in CHD as compared with age‐matched controls (Figure 1F–G). Furthermore, we found a significant increase in double‐negative CD27 and CD28 CD8 T cells (Figure 1H) and in the expression of CD57 on CD8 T cells (Figure 1I) in CHD, which is similar to the findings of Elder and colleagues on CD8 T cells in adult CHD [33]. While CD57 expression is associated with a replicative senescence state, the loss of CD27 and/or CD28 molecules is associated with a senescent phenotype in T cells [34]. In summary, children with CHD who underwent congenital heart surgery within the first year showed a perturbed T cell compartment with an increase of memory T cells, senescent‐like T cells, and reduced RTE.

### 3.2 Single Cell Transcriptomics Reveals Lower Naïve and Higher Effector αβ T Cells in CHD

**FIGURE 2:**
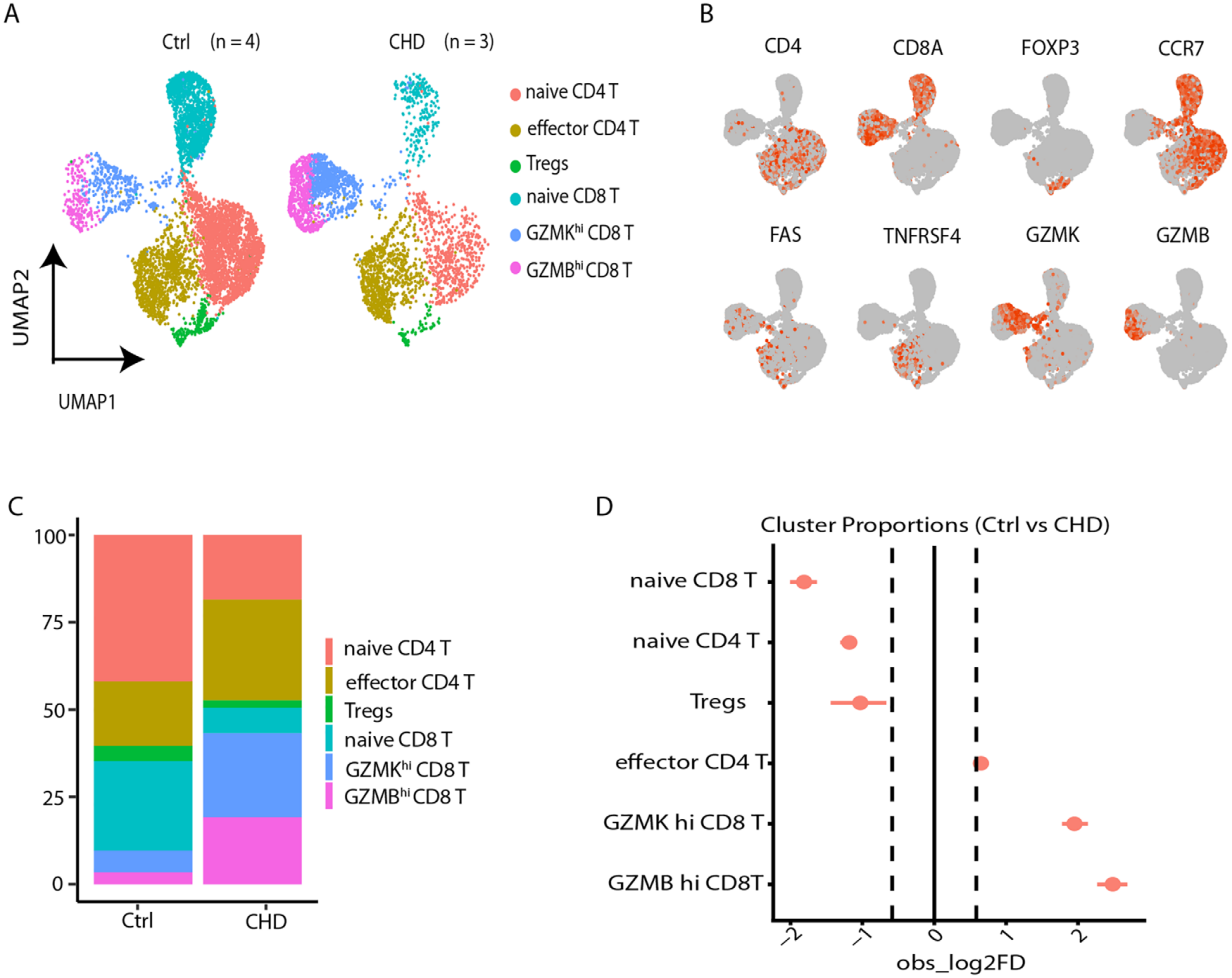
scRNA‐seq of T cells in children with CHD. (A) UMAP visualization of identified clusters from scRNA‐seq of FACS‐sorted αβ T cells from 5‐ to 12‐year‐old children who received thymectomy within a year after birth (CHD, *n* = 3) and control children (GSM8443049), colored by cluster. (B) Canonical cell markers were used to label clusters by cell identity as represented in the UMAP. (C) Stacked bar plot showing the percentage of each cluster's contribution per condition. (D) Relative differences in cell proportions for each cluster between the Ctrl and CHD. Clusters colored red have an FDR < 0.05 and abs | Log2 fold difference | > 0.58 (permutation test; *n* = 10,000).

To understand the underlying mechanisms of the responsiveness of the T cells in CHD children, a single‐cell transcriptome (scRNA‐seq) analysis was performed. For this, we generated new single‐cell transcriptomics data to investigate the immune profiles of αβ T cells from three patients with CHD (Table 2). For non‐CHD controls, we took advantage of a dataset of four healthy children from our previous study (GSM8443049) [23]. After filtering out low‐quality droplets, a TCR expression gene module score was used to isolate αβ T cells from total T cells, namely the exclusion of γδ T cells, as described by Song et al. [30]. A total of 6025 αβ T cells from the control group and 3204 αβ T cells from CHD children were included for downstream analysis. Using graph‐based clustering of uniform manifold approximation and projection (UMAP) and unsupervised clustering, the expression of signature genes for each subset was used from the differential gene expression analysis to annotate six major cell clusters (Figure 2A). The clusters were annotated according to the expression of canonical gene markers for naïve and effector CD4 and CD8 αβ T cells and the top 10 differentially expressed genes (DEGs) (Figure 2B, Figure S2E). Thus, the clusters were divided into naïve CD4 T cells (*SELL*+*CCR7*+), effector CD4 T cells (*TNFRSF4*+), regulatory T cells (*FOXP3*+), naïve CD8 T cells (*SELL*+*CCR7*+), GZMK^hi^ CD8 T (*GZMK*+), and GZMB^hi^ CD8 T cells (*GZMB*+).

**TABLE 2:** Summarized characteristics of children with CHD postsurgery and controls used for single‐cell RNA‐sequencing.

Characteristic	Children with CHD (*n* = 3)	Ctrl (*n* = 4)
Age in years at sample collection: Median (IQR)	6.92 (0.54)	7.75 (5.4)
Age in months at time point of cardiac surgery involving (partial) removal of thymic tissue: Median (IQR)	7 (2.00)	NA
Sex (M/F)	1/2	2/2

Abbreviations: CHD: congenital heart disease, Ctrl: age‐matched controls, NA: not applicable, M: male, F: female.

Both CHD and age‐matched controls (Ctrl) contributed to all six clusters (Figure S2F). To reveal the differences in cluster composition between CHD children and control children, the relative percentage of the six cell clusters in each condition was calculated (Figure 2C). Moreover, we performed a single‐cell proportion test that facilitates the analysis of the difference between the proportions of cells in clusters between two scRNA‐seq samples [32], and revealed a reduction of naïve CD8 and CD4 T cell clusters in children with CHD (Figure 2D). Altogether, the transcriptional analysis revealed a higher proportion of effector CD8 and CD4 T cells in children with CHD.

### 3.3 Differential Gene Expression Profiles Show High Activation States of T Cells in Thymectomized CHD Children

**FIGURE 3:**
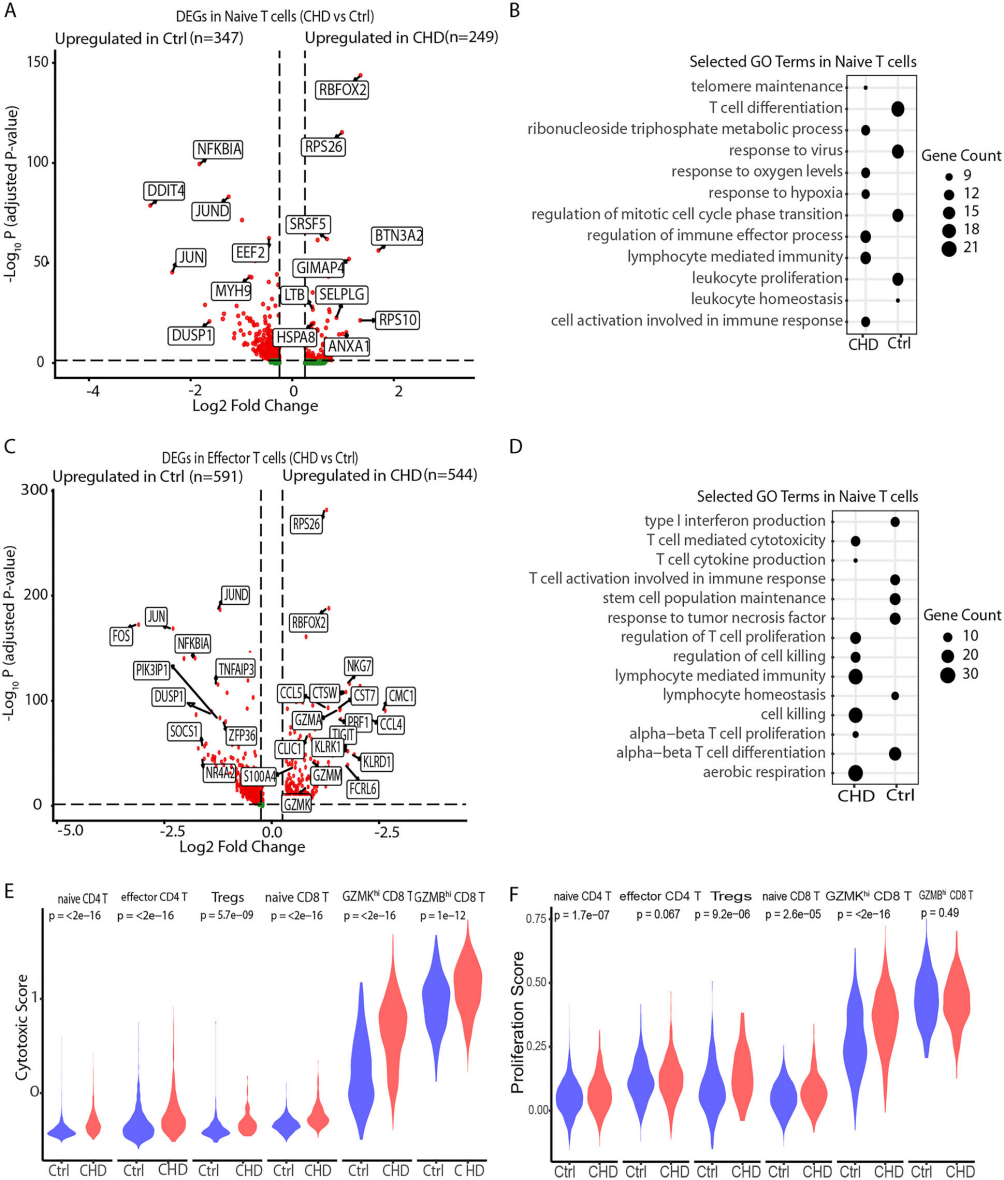
Increase in the expression of effector molecules in T cells of children with CHD. (A, C) Volcano plot showing the differentially expressed genes in naïve T cells (A) and effector T cells (C) between CHD and Ctrl. Each point represents a gene, with log_2_ fold change (*x*‐axis) indicating the magnitude of expression change and −log_10_ adjusted *p*‐value (*y*‐axis) representing statistical significance. Red points indicate significantly significant genes (adjusted *p*‐value < 0.05, |log_2_FC| > 0.25), while green points represent nonsignificant genes. Selected top DEGs are labelled. (B, D) Gene Ontology (GO) of significantly upregulated genes in naïve T cells (B) and Effector T cells (D) in Ctrl and CHD. Each point represents a GO term, with its size reflecting gene count. (E) Violin plot of the single‐cell gene signature module score for cytotoxicity. (F) Violin plot of the single‐cell gene signature module score for proliferation. The cytotoxic score was computed based on *CCL5, CCL4, GZMA, GZMB, GZMH, GZMK, CXCR6, NKG7, GNLY, KLRK1, KLRD1, KLRF1, KLRC1, KLRB1*, and *KLRG1* and the proliferation score was based on *CCND3, VAV3, HLA‐G, TSPAN32, FCRL3, SOS2, HLA‐DPB1, ANXA1, CD81, FYN, HMGB1, GSTP, SH2D2A, TNFRSF1B, MSN, IRF1, HLA‐DPA1, CD300A, NFATC2, CCL5, CD74, ZAP70, SELENOK, CD3E, SPN, CBLB, RASGRP, PSMB10, MAPK1, HLA‐E, TYROBP, CEBPB, PTPRC, RASAL3, ITCH, HLA‐DRB1, TNFRSF14, FOSL2, PKN1, BAX, BST2, BTN3A1, RC3H1, PURA,NCK1, TCIRG1, CD320, MYD88, ADA, HLA‐A, TBK1, ZNF335, CTPS1, TRAF6, LYN, TNFSF14, TYK2, ABL1, SLC7A1*.

Next, we investigated the transcriptomic changes of T cells in children with CHD postthymectomy. Both naïve CD4 T and naïve CD8 T shared similar upregulated and downregulated genes (Figure S3A,B). Therefore, we performed differential gene expression (DEG) analysis of all naïve T cells between CHD and Ctrl, identifying 249 upregulated genes in CHD and 347 upregulated genes in Ctrl (Figure 3A). Among those genes that are upregulated in the CHD group are genes associated with survival, biogenesis, and homeostasis (*RPS26* and *RPS10*) [35], and *RBFOX2*, with a well‐characterized role in alternative splicing regulation of pre‐mRNAs and shown to be a key stress sensor in heart disease [36, 37]. GIMAP4, a member of the GTPase of the immunity‐associated protein (Gimap) family, has also been associated with T cell‐apoptosis and regulates secretion of cytokines in early differentiating human CD4+ Th lymphocytes [38, 39]. Furthermore, butyrophilin subfamily 3 member A2 (BTN3A2) was upregulated in CHD, known to serve as a crucial mediator in immune activation and has been shown to be mainly involved in T‐cell receptor interaction and the nuclear factor‐κB (NF‐κB) signaling pathway [40]. On the other hand, downregulated genes are associated with T cell development, proliferation, and survival, such as the *NFKBIA* gene (IκBα), which is involved in the NF‐κB signalling pathway, and its deficiency might impair the activation and immune function of T cells [41]. In addition, *FOS, JUN*, and *JUND*, which belong to AP‐1 family transcription factors, known to have a central role in transducing TCR‐driven effector programs and are involved in mediating many biological processes such as proliferation, differentiation, and cell death, were downregulated in the CHD group (Figure 3A; Figure S4A) [42]. Further investigation of the biological processes that are associated with these DEGs revealed telomere maintenance, cell activation, response to oxygen levels upregulated in CHD, and processes such as T cell differentiation, proliferation, and response to virus were upregulated in Ctrl (Figure 3B).

Next, the DEG analysis focused on effector T cells. Proinflammatory genes such as *IL32*, *CCL5*, *S100A4*, and *S100A11* are upregulated in effector CD4 T cells in CHD, whereas cytotoxic genes (*PRF1*, *KLRD1*, *NKG7*, *CST7*, *GZMA*, *EOMES*, *XLC2*) and inhibitory genes (*TIGIT* and *CD160*) were upregulated in effector CD8 T cells (GZMK^hi^ and GZMB^hi^ CD8 T cells) (Figure S3C,D). Furthermore, DEG analysis of all effector T cells, comprising CD4 and CD8 T cells, between CHD and Ctrl was performed. The analysis identified 544 upregulated and 591 downregulated genes (Figure 3C). Among the genes that are upregulated are chemokines (*CCL4, CCL5*) produced by activated T cells associated with chemotaxis and other patho/physiological processes [43], genes associated with cytotoxicity (*PRF1, GZMA, GZMK, GZMM, NKG7*), and *S100A4*, a metastasis‐promoting gene, a member of the S100 family, recently implicated in various inflammation‐associated diseases. *S100A4* is also known for cell survival, motility, regulation of angiogenesis, and invasion [44, 45, 46]. Similarly, the downregulated genes include the AP‐genes and *PIK3IP1* (PI3K‐interacting protein‐1), also known as the transmembrane inhibitor of PI3K, whose loss in T cells leads to an increase in T cell activation (Figure 3C) [47]. Further investigation of the biological processes that are associated with these DEGs revealed T cell‐mediated cytotoxicity, cytokine production, proliferation, and cell killing in CHD and type 1 interferon response, T cell activation, stem cell population maintenance, lymphocyte homeostasis, and alpha beta T cell differentiation in Ctrl (Figure 3D).

Focusing on the functional role of the identified clusters, there was an increase of *GZMA*, *GZMH*, *NKG7*, *GNLY*, and *PRF1* on CD8 T cells and TNFRSF4 (encoding *CD134*) on effector CD4 T cells in children with CHD (Figure S4B). A cytotoxicity module score computed in the respective six clusters suggests an overall increased cytotoxicity in children with CHD (Figure 3E). Notably, the effector clusters showed increased senescent‐associated genes and type 1 transcription factors in CHD (Figure S4C). Furthermore, a proliferation gene module score showed a higher proliferative capacity in naïve CD4, naïve CD8, and GZMK^hi^ CD8 T cells (Figure 3F), and a recent thymic emigrant score on the naïve T cells compartment showed less thymic output in CHD compared with the control (Figure S4D). This could be due to homeostatic proliferation, as the immune system has the ability to maintain T cells in the absence of thymic production or antigenic stimulation [18, 48]. In summary, the single‐cell RNA sequencing revealed the functional state of T cells in children with CHD and points to the presence of granzyme‐producing T cells in children with CHD.

### 3.4 Higher Effector Functions of T Cells in Children With CHD

**FIGURE 4:**
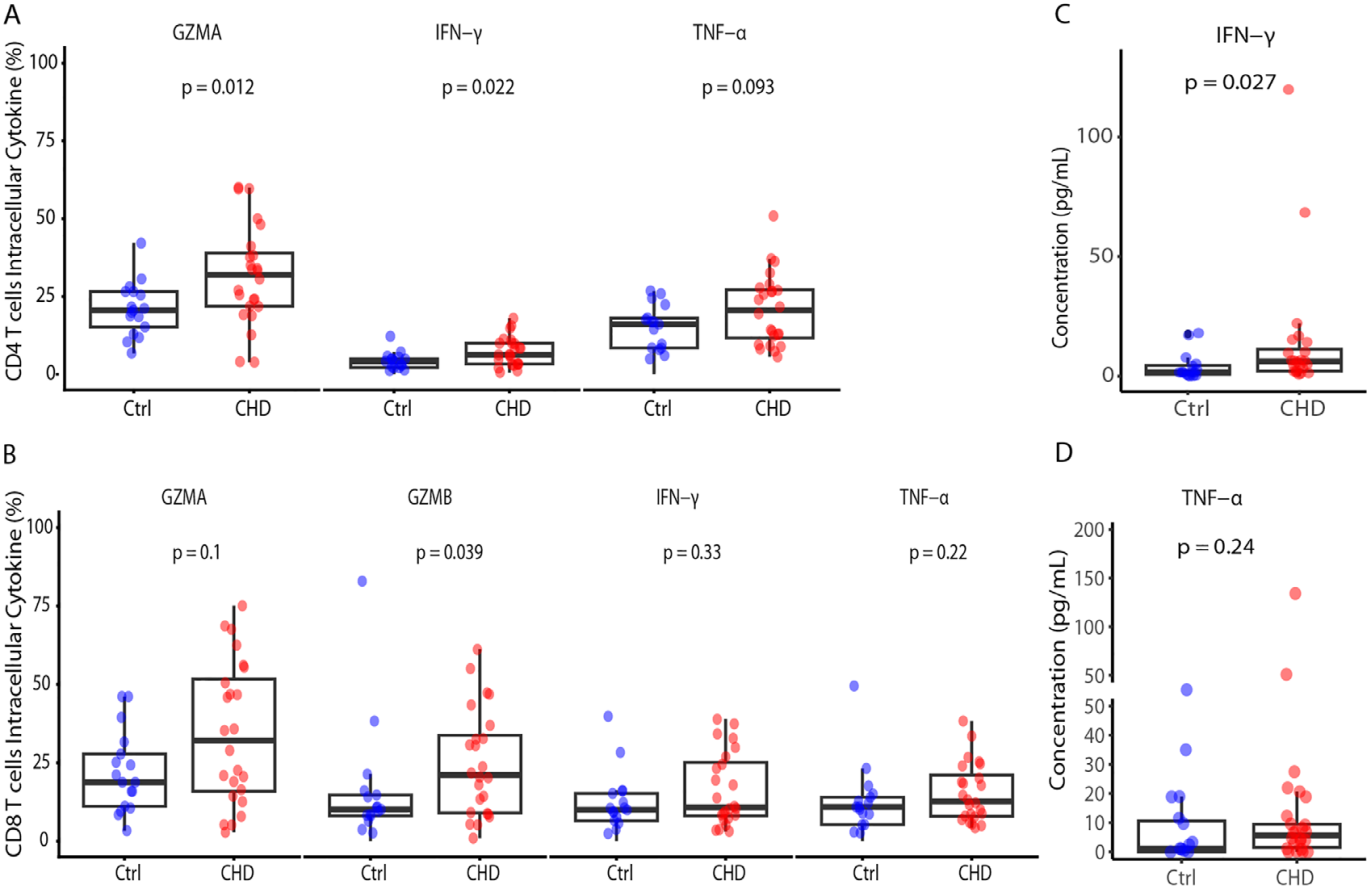
Increased effector functions of T cells and a pro‐inflammatory state in children with CHD. (A) Percentage of intracellular cytokine detection of total CD4 T cells among CHD and age‐matched controls after 3 h PMA/Ionomycin stimulation. (B) Percentage of intracellular cytokine secretion by total CD8 T cells among CHD and age‐matched controls after 3 h PMA/Ionomycin stimulation. (C, D) Cytokine concentration (C) IFN‐γ (D) TNF‐α, from the plasma of CHD and Ctrl. Statistical analyses were performed using the Mann–Whitney *U* test, and a *p*‐value < 0.05 was considered significant. Horizontal bars indicate median values. Each dot represents one donor.

Next, we investigated the functionality of T cells. For this reason, an in vitro stimulation with phorbol myristate acetate (PMA) and ionomycin of PBMCs from children with CHD (*n* = 24) and age‐matched controls (*n* = 17) was performed, following measurement of intracellular GZMA, GZMB, IFN‐γ, and TNF‐α within CD4 and CD8 T cells (Figures S5A–C and S6A–D). For CD4 T cells, there was a significant increase in GZMA and IFN‐γ production, but not for TNF‐α production, in the CHD group as compared with Ctrl (Figure 4, A; Figure S5). For CD8 T cells, there was a particularly significant increase in GZMB production, but not GZMA, IFN‐γ, and TNF‐α production, in the CHD group as compared with the Ctrl group (Figure 4B; Figure S6). In addition, part of the measured samples (*n* = 8 per group) were stimulated with anti‐CD3/anti‐CD28 for 6 h. In that case, GZMA production by CD4 and CD8 T cells was higher in the CHD group as compared with the Ctrl group (Figure S7A,B). However, other cytokines measured did not reach statistical significance, except for IFN‐γ, which was higher in Ctrl CD8 T cells (Figure S7B).

In addition, we studied pro‐inflammatory cytokines in the plasma of the children with CHD (*n* = 24) and controls (*n* = 16), since IFN‐γ has long been associated with inflammation and autoimmune disease [49]. Higher levels of IFN‐γ in the plasma of children with CHD were observed (Figure 4C), which is considered a pro‐inflammatory cytokine that directs T helper (Th)1 cell differentiation [50]. Though we observed no significant difference in TNF‐α (Figure 4D), an unsupervised clustering approach of all the measured cytokines, including MCP‐1, IL‐6, IFN‐α2, IL‐33, IL‐17A, IL‐1β, IL‐8, IL‐10, IL‐23, showed a low‐grade inflammatory state in some children with CHD (Figure S7C). In summary, the abundance of more effector T cells in children with CHD and cardiac surgery with thymectomy may lead to higher granzyme and cytokine production, which suggests a pro‐inflammatory state in those children with CHD.

### 3.5 Single‐Cell RNA Sequencing Reveals Higher Expression of Inhibitory Receptors in Children With CHD

**FIGURE 5:**
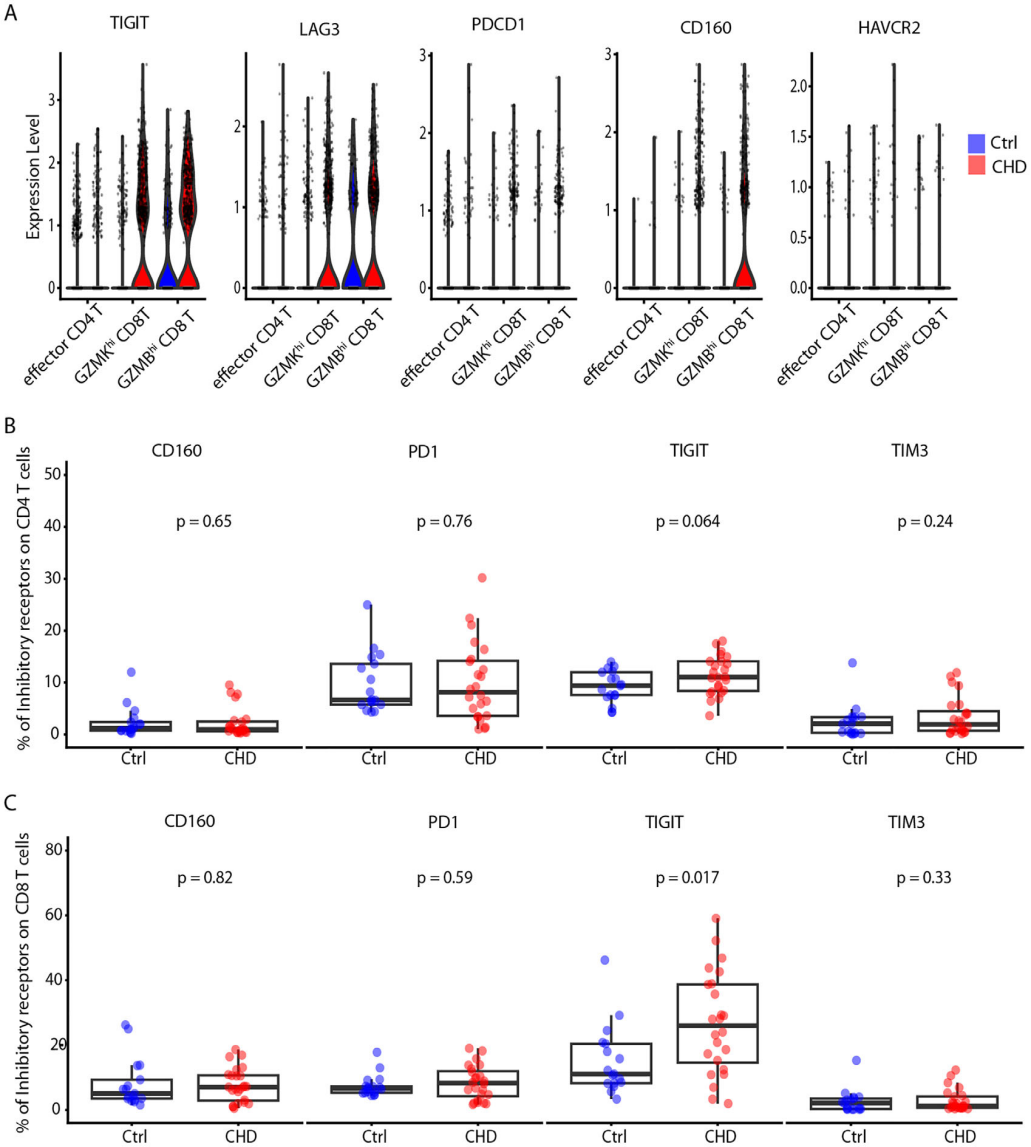
Expression of Inhibitory Receptors in children with CHD. (A) Violin plot showing the average expression of *TIGIT, LAG3, PDCD1, CD160*, and *HAVCR2* between CHD and Ctrl. (B) Percentage of Inhibitory receptors on CD8 T cells among CHD and Ctrl after 3 h PMA/Ionomycin Stimulation. (C) Percentage of Inhibitory receptors on CD8 T cells among CHD and age‐matched controls after 3 h PMA/Ionomycin Stimulation. Statistical analyses were performed using the Mann–Whitney *U* test, and a *p*‐value <0.05 was considered significant. Horizontal bars indicate median values. Each dot represents one donor.

Next, we investigated whether the higher memory phenotypes and higher effector functions seen in children with CHD could lead to the upregulation of inhibitory receptors. Also, continuous antigenic stimulation during chronic infections has been shown to cause immune exhaustion [34, 51]. Thus, we investigated expression levels of inhibitory receptors in our single‐cell transcriptome data. There was higher expression of *TIGIT*, *LAG3*, *PDCD1*, and *CD160* in CHD and no difference in the expression of *HAVCR2* (TIM3) between CHD and Ctrl in the effector T cell clusters (Figure 5A). In addition, *CTLA4*, *CD244*, and *BTLA* were investigated, which showed the highest expression levels on effector clusters (effector CD4, GZMB^hi^, and GZMK^hi^ CD8 T cell) (Figure S8A). Next, we measured CD160, LAG3, PD1, TIGIT, and TIM3 expression on CD4 and CD8 T cells after 3 h PMA/ionomycin stimulation by flow cytometry (Figure S8B, C). Elevated expression levels of TIGIT on CD8 T cells were evident (Figure 5B). However, for the majority of measured receptors, there was no significant difference in the expression among CD4 T cells (Figure 5C) and CD8 T cells (Figure 5B). In summary, investigation of transcriptomes and surface proteins of single cells reveals higher expression of TIGIT on effector CD8 T cells in children with CHD who underwent cardiac surgery with thymectomy within the first year of life.

### 3.6 Treg Cells Display a Higher Expression of Suppressive Genes in Children With CHD

**FIGURE 6:**
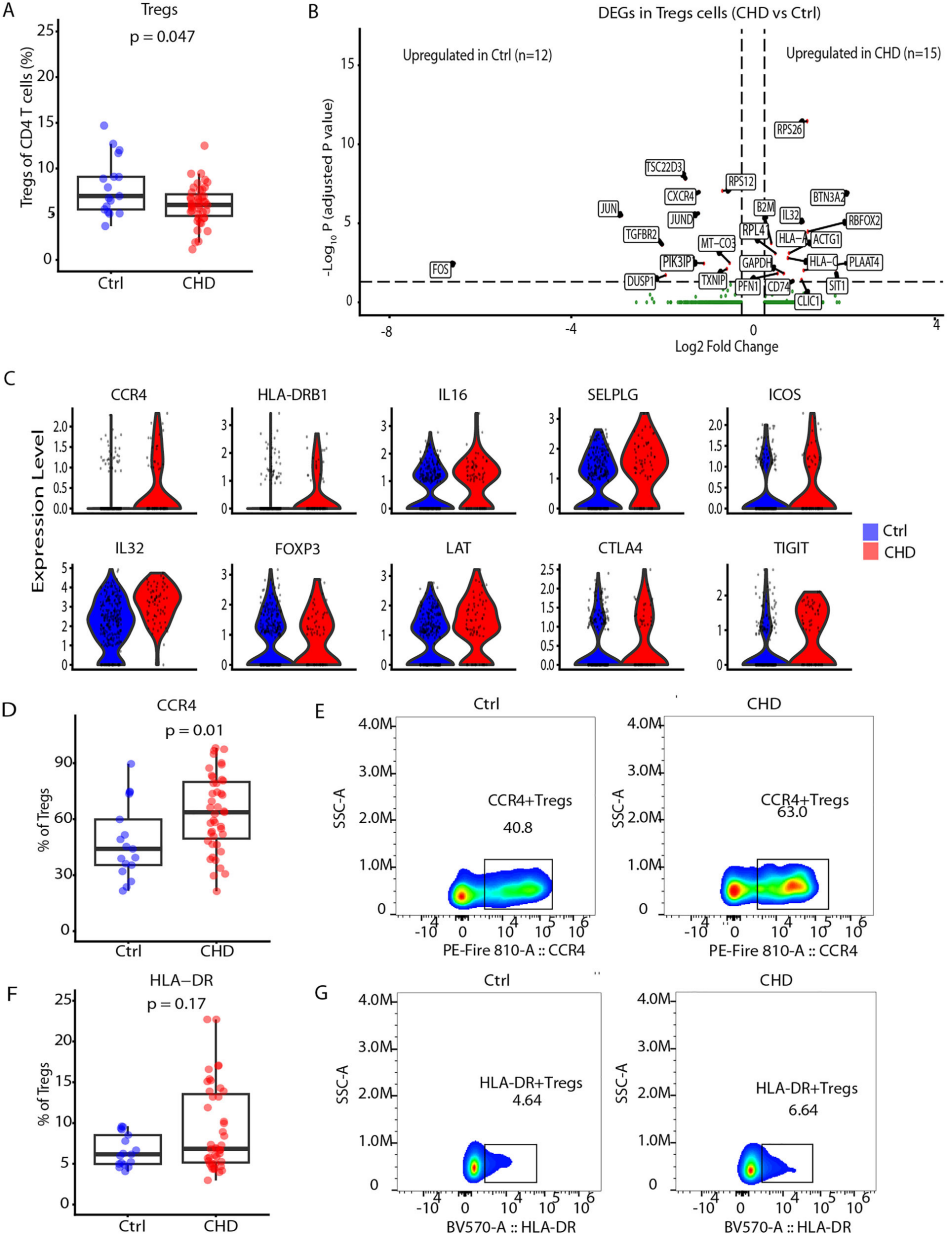
Reduced Treg cells with higher suppressive genes in children with CHD. (A) Boxplot showing the percentage of regulatory T cells within CD4+ T cells (CD45RA^low^ CD25^+^). (B) Volcano plot showing the differentially expressed genes in Tregs between CHD and Ctrl. Each point represents a gene, with log_2_ fold change (*x*‐axis) indicating the magnitude of expression change and −log_10_ adjusted *p*‐value (*y*‐axis) representing statistical significance. Red points indicate significantly significant genes (adjusted *p*‐value < 0.05, |log_2_FC| > 0.25), while green points represent nonsignificant genes. (C) Violin plot showing the average expression of *CCR4, HLA‐DRB1, IL16*, and *SELPLG* between CHD and Ctrl. (D) Boxplot showing the percentage of CCR4+ Treg cells among all Treg cells. (E) Representative flow cytometric gating strategy to define CCR4 expression. (F) Boxplot showing the percentage of HLA‐DR+ Treg cell subsets within Treg cells. (G) Representative gating strategy to define HLA‐DR expression.

Next, we asked if regulatory T (Tregs) cells, known to have a potent immunosuppressive capacity and critical for the prevention of autoimmunity, could contribute to preventing inflammation in these patients [52]. First, the abundance of regulatory T cells defined by CD25 and CD127 was measured by flow cytometry (Figure S1A), and a significant reduction in the Tregs among CD4 T cells was observed (Figure 6A). Thus, we investigated the transcriptional changes among the Tregs in our study group and performed differential gene expression (DEG) analysis of Tregs between CHD and Ctrl, identifying 15 upregulated and 11 downregulated genes (Figure 6B). Again, AP‐1 genes (*FOS, JUN, JUND*) were downregulated in CHD Tregs. Also, the gene *CXCR4*, known for being critical in the homing of T regulatory (Treg) cells to the bone marrow (BM) to resolve inflammation, was downregulated in CHD Tregs [53, 54]. Interestingly, among the upregulated genes in CHD was the proinflammatory cytokine interleukin 32 (IL32), shown to induce Foxp3 expression in CD4+ T cells to suppress the tumor immune response [55]. We further investigated the expression of other genes associated with suppressive capacity of Tregs, such as *FOXP3*, *ICOS*, *IL32*, *LAT*, *CTLA4*, *TIGIT*, *CCR4*, *HLA‐DRB1*, *IL16*, and *SELPLG* [52, 56, 57, 58, 59], which were highly expressed in the CHD group as compared with the Ctrl group (Figure 6C). Additional flow cytometric analysis showed a significant increase in CCR4+Tregs (Figure 6D,E). For HLA‐DR, there was no significant difference in HLA‐DR+ Tregs, though with higher interindividual variability (Figure 6F,G). In summary, in the children with CHD, there was a reduced Treg cell abundance, and those showed higher expression of suppressive genes.

## 4 Discussion

CHD is the most common congenital anomaly, involving structural abnormalities of the heart or intrathoracic great vessels [1]. The prevalence of CHD births has increased over the last century to an estimated 9 out of 1000 live births worldwide, amounting to 1.35 million CHD births annually [60]. Improved diagnostics and therapeutic interventions have led to a 97% survival rate among children into adulthood [11]. The growing adult population with CHD underscores the necessity for research focusing on the long‐term consequences of CHD treatment.

Recent population‐based studies indicate that infant cardiac surgery with a potential concomitant thymectomy to ensure adequate surgical access leads to limited immune competence and is associated with an increased risk of morbidity, including infections, cancers, autoimmune diseases, and atopy [12]. Studies investigating the immune status of these children have provided evidence of immune dysregulation, including reduced T cell populations, reduced proportion of recent thymic emigrants, and accumulation of memory T cells [18, 21, 23, 61, 62, 63]. Notably, Bremer and colleagues observed thymic atrophy (a condition in which the thymus shrinks and loses function) in patients with complex cyanotic CHD even before the first cardiac surgery [64]. In this study, we only included patients with various types of CHD who underwent congenital heart surgery in the neonatal or infant period, but not those without surgery. We observed a reduced thymic activity, as determined by CD31 expression on naïve CD4 T cells [65]. Currently, there are no reliable surface markers associated with RTEs in the naive CD8+ T cell pool, as CD31 or PTK7 are only used on naïve CD4 T cells and not on naïve CD8 T cells. Notably, a recent finding by Bohacova et al. [66] suggested CD38 as a marker for RTE in both CD4 and CD8 expression. However, at the time of sample acquisition, these findings had not yet been published and were therefore not included. Importantly, our scRNA‐seq data also showed reduced thymic output score for both CD4 and CD8 in thymectomized children.

Transcriptional analysis suggests that the remaining αβ T cells possess a higher proliferative capacity, thereby compensating for the lower thymic activity. The maintenance of T cell numbers in the periphery is mediated by distinct homeostatic mechanisms that ensure the preservation of naive and memory T cells [8, 67]. The direct association between increased turnover and decreased frequency of naive T cells has been shown to be particularly active in lymphopenic hosts, such as elderly and thymectomized patients [48, 67]. Conversely, the immune system generates memory phenotype cells with increasing T cell receptor repertoire complexity by a unique process termed spontaneous proliferation [67]. Moreover, an increase in memory T cells may be associated with immune senescence. This is because memory T cells lose their quiescent state with time and acquire intrinsic defects through repeated proliferation, a process known as replicative senescence. This phenomenon has been shown to lead to compromised immunity, chronic inflammation, and inflammatory disorders, including autoimmune diseases, HIV‐associated immune reconstitution inflammatory syndrome, Omenn syndrome, and bare lymphocyte syndrome over time as a result of T cell dysregulation [6, 67, 68]. The T cells that are produced from this extrathymic process undergo enhanced proliferation and cytokine secretion more readily than classic T cells, but are prone to apoptosis [69]. Thus, one of the main focuses of this study was to investigate the functionality of T cells in children with CHD who underwent congenital heart surgery in early life.

To date, a variety of agents have been employed to assess the proliferative capacity and cytokine production of T cells. These include mitogens such as phytohaemagglutinin (PHA) [21, 62] and concanavalin A [21], anti‐CD3/CD28 [23, 24, 63], HMBPP [23], cytokines such as recombinant human IL‐2 (rhIL‐2) and rhIL‐7 [22], and specific antigens of tetanus toxins, measles virus [70], CMV, and EBV [22]. However, existing literature yields conflicting results on proliferation capacity and cytokine production of T cells without a detailed analysis [20, 21, 24, 62, 71].

Our single‐cell transcriptional analyses revealed an increase in effector states in αβ T cells, as defined by an increase in the expression of genes associated with activation and cytotoxicity. Consistent with these observations, in vitro stimulation of T cells revealed enhanced granzyme A and B production in CD8 T cells. These findings suggest that 5 to 10 years following congenital heart surgery with complete or partial thymectomy, the T cell compartment maintains its functionality and is capable of mediating immune responses to infections.

Children with CHD exhibited a slightly higher abundance of pro‐inflammatory cytokines. This finding may possibly indicate a low‐grade pro‐inflammatory state, where the immune system is persistently or aberrantly activated. This has previously been shown in a study that reported elevated serum levels of IL‐1β, IL‐8, and eotaxin in young adults who underwent congenital heart surgery with thymectomy within the first 2 weeks of life [22]. As inflammatory markers may be predictive of inflammatory diseases [72], the production of higher levels of pro‐inflammatory cytokines may compromise adaptive immunity in CHD patients [64] and contribute to the severe clinical course of diseases and other complications in these children later in life [14, 73, 74, 75]. Further investigation in larger patient cohorts, and also how this may relate to the CHD disease itself, is therefore warranted.

To better understand the underlying mechanisms that control the functionality of T cells within children with CHD, the expression of co‐inhibitory receptors was studied. An increase in the expression levels and frequencies of co‐inhibitory receptor TIGIT on CD8 T cells was observed in children with CHD as compared with the control group. TIGIT has been identified as a receptor on T cells to control excessive proliferation and function [76], and might be important to regulate the functionality of effector CD8 T cells in children with CHD. In general, co‐inhibitory receptors on the surface of cytotoxic cells are associated with maintaining tolerance within a feedback mechanism, as they act as a switch‐off in the immune system to prevent auto‐reactivity and excessive functionality [77]. At the same time, the expression of inhibitory receptors can also be associated with T cell exhaustion. Although studies have reported high expression of the inhibitory receptor PD‐1 in children with CHD [23, 33], we did not observe states of T cell exhaustion in our transcriptome and functional data. Our data suggest that the T cell pool has enhanced functionality due to a lower number of naïve T cells, and the remaining T cells are in a state of final differentiation and activation. The latter presumably leads to the upregulation of inhibitory receptors, such as TIGIT, as a feedback mechanism to prevent excessive immune activation [78]. Consistent with this idea, and despite the low number of Treg cells in this study cohort, the remaining Treg cells displayed higher suppressive genes, which might be an additional compensatory mechanism to control the proinflammatory state in these children. Though no functional in vitro experiments were performed in this study, and it remains unclear whether in vitro assays of Treg function recapitulate Treg function in vivo [79], we believe that the expression of those genes could support controlling the overactivation of the immune system owing to the long‐standing role of regulatory T cells suppressing inflammatory responses in a variety of biological contexts [52, 59, 80]. The downregulation of CXCR4 in Tregs in this group warrants further investigation, as recent evidence shows that CXCR4 surface expression is critical for Tregs to home into bone marrow and resolve inflammation [53, 54].

In summary, this study investigated the functionalities of T cells from transcriptomics to the protein level and points to a higher granzyme production capacity of CD8 T cells and an increased expression of TIGIT on CD8 T cells in children with CHD. This may reflect a dysregulation of T cell homeostasis with an ongoing activation of the T cell population. It remains to be seen whether the sustained and chronic activation over time may lead to a progressive loss of T cell effector function, resulting in exhausted or dysfunctional T cells in adult CHD patients.

## Author Contributions

Study concept and design: S.R., M.B.; recruitment and sample collection: Y.E.A., A.C., S.K., C.K., A.H., C.J., A.J., M.B.; experiments: Y.E.A., T.Y., A.J., R.G.J.; data analysis: Y.E.A.; data interpretation: Y.E.A., C.K., R.F., M.B., S.R.; data visualization: Y.E.A.; statistics: Y.E.A.; writing of the manuscript: Y.E.A.; finalized the draft: S.R., M.B. The final version of the manuscript was read and approved by all authors.

## Funding

Hannover Biomedical Research Schools and the Center for Infection Biology (ZIB) supported Y.E.A. The German Research Foundation Deutsche Forschungsgemeinschaft (DFG) under Germany's Excellence Strategy supported the work, EXC 2155 RESIST, Project ID 390874280 to R.F. and S.R.; and the DFG‐funded research group FOR2799 Project ID RA3077/1‐2 and the project RA3077/3‐1 to S.R. and Kinderherzen Fördergemeinschaft Deutsche Kinderherzzentren e.V. to M.B. and S.R.

## Conflicts of Interest

The authors declare no conflicts of interest.

## Supporting information




**Supporting File**: eji70209‐sup‐0001‐SuppMat.pdf.

## Data Availability

Raw and processed scRNAseq data are accessible under NCBIs Gene Expression Omnibus (https://www.ncbi.nlm.nih.gov/geo/query/acc.cgi?acc=GSE299265).
