# Supplementary material for: Sustained Effector Functions and Memory Accumulation of αβ T Cells in Children With Congenital Heart Disease
Source: Eur J Immunol. 2026 May 13;56:e70209. doi: 10.1002/eji.70209 (PMC13169477; doi:10.1002/eji.70209)
Supplement: Supplementary file 1 — Supporting File: eji70209‐sup‐0001‐SuppMat.pdf. [file EJI-56-e70209-s001.pdf]

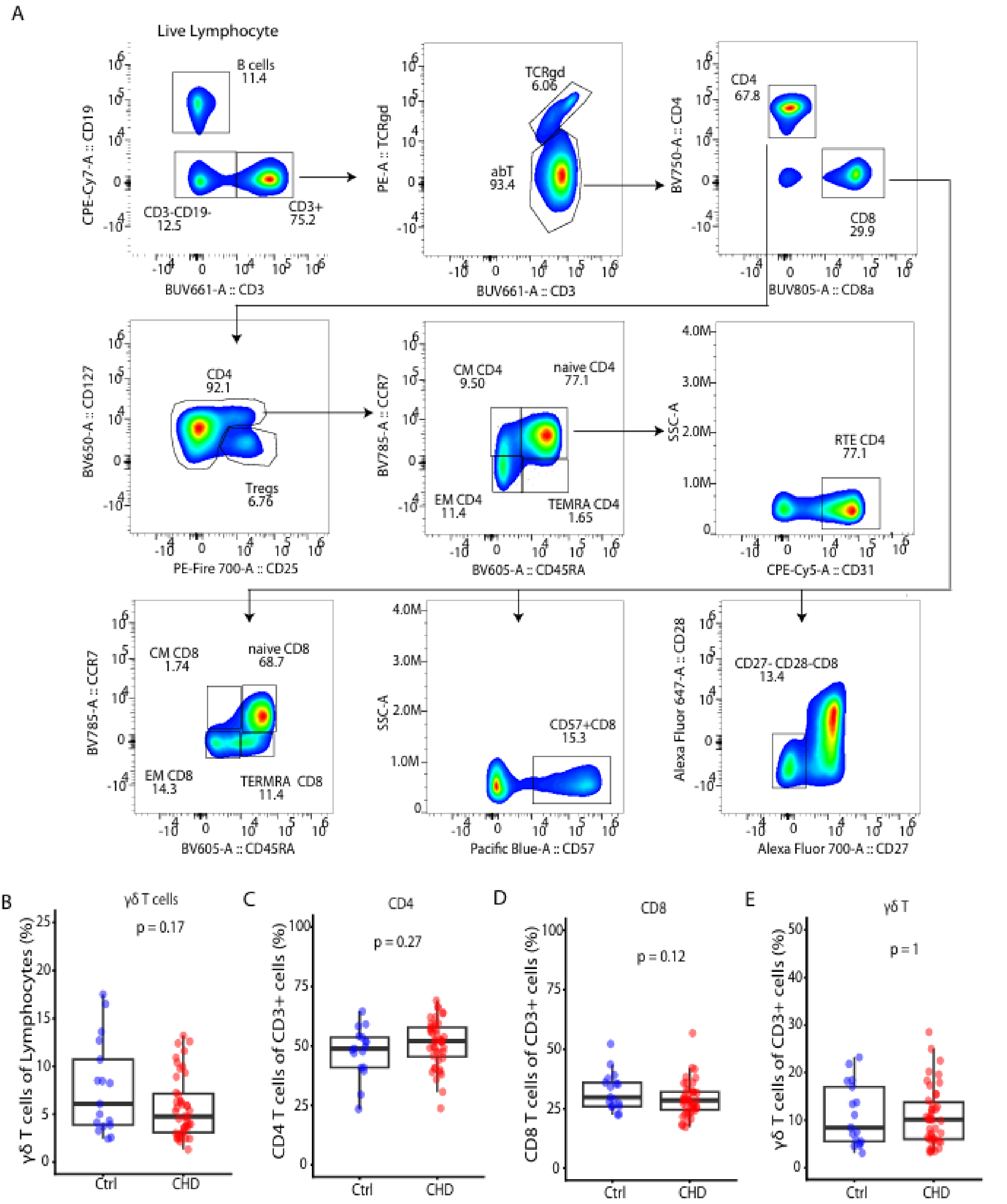

FIGURE S1: Gating Strategy and Proportion of T cells

A) Gating strategy on a representative sample showing the different T cell subsets and expression of markers on the subsets. **B**) Boxplot showing the percentage of γδT cells within live lymphocyte. **C**) Percentage of CD4 T cells within CD3+ T cells. **D**) Percentage of CD8 T cells within CD3+ T cells. **E**) Percentage of γδT cells within CD3+ T cells

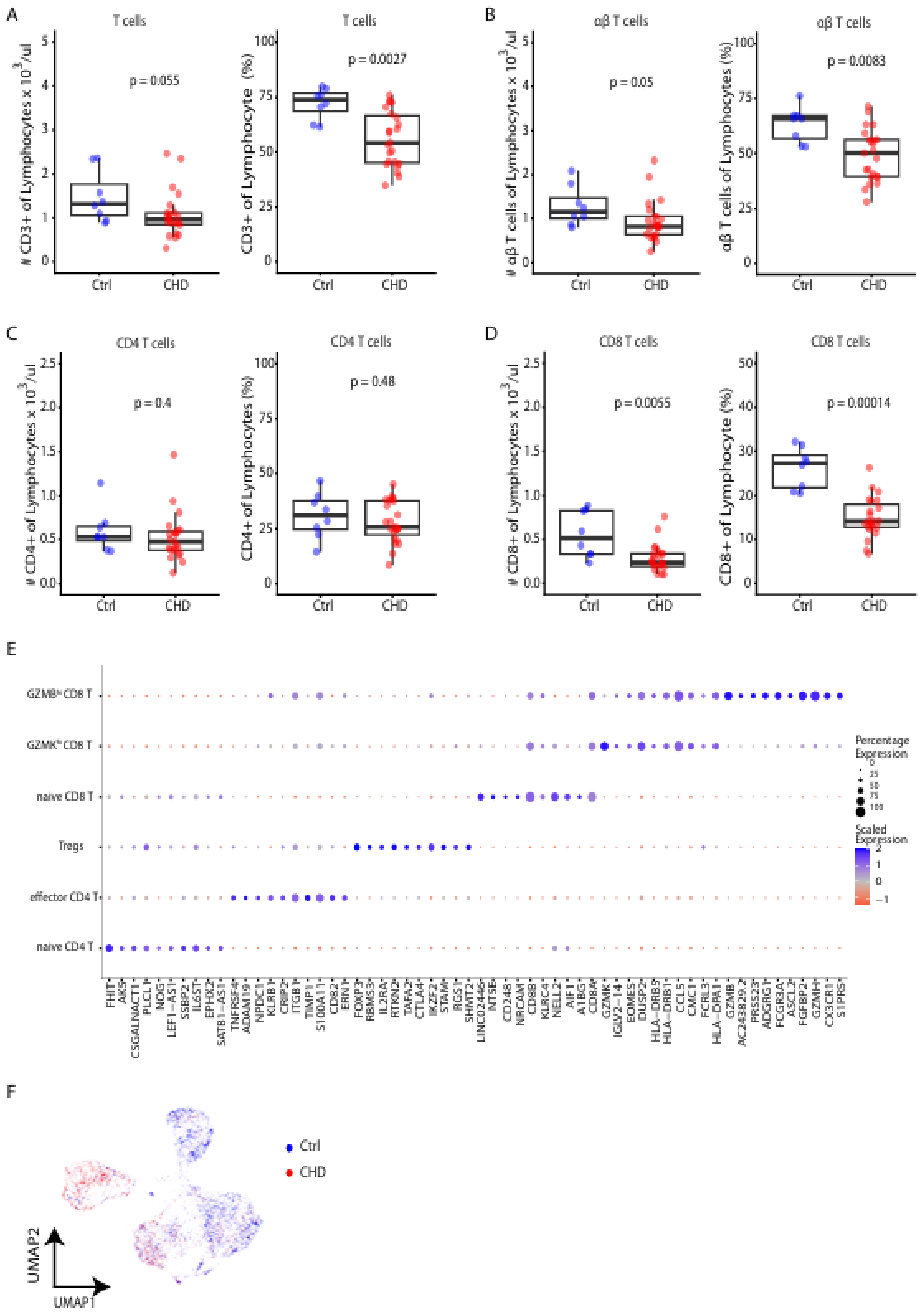

FIGURE S2: Cell Count of T cells, differentially expressed genes and cell contribution per condition

A) Boxplot showing the cell count (Left panel) and percentage (Right panel) of CD3 T cells within live lymphocytes. **B**) Boxplot showing the cell counts (Left panel) and percentage of αβ T cells within live lymphocytes. **C**) Boxplot showing cell count (Left panel) and percentage (Right panel) of CD4 T cells within alive lymphocytes. **D**) Boxplot showing the cell count (Left

panel) and percentage (Right panel) of CD8 T cells within live lymphocytes. **E**) The dot plot shows the top 10 up-regulated DEGs (columns) per cluster (rows). Gene expression values were scaled to a log2 fold change (logFC). Dots are colored by average logFC and sized by percentage of cells per cluster that ex pressed this gene (pct.exp). DEGs in this study are defined as follows: (i) absolute value of average logFC ≥ 0.25 and (ii) detected on at least 25% of cells from at least one cluster (min.pct = 0.25). **F)** UMAP visualization of identified clusters from scRNA- seq of FACS-sorted αβ T cells from 5- to 12-year-old children who received thymectomy within 1 year after birth (CHD, n = 3) and control children(GSM8443049), colored by condition.

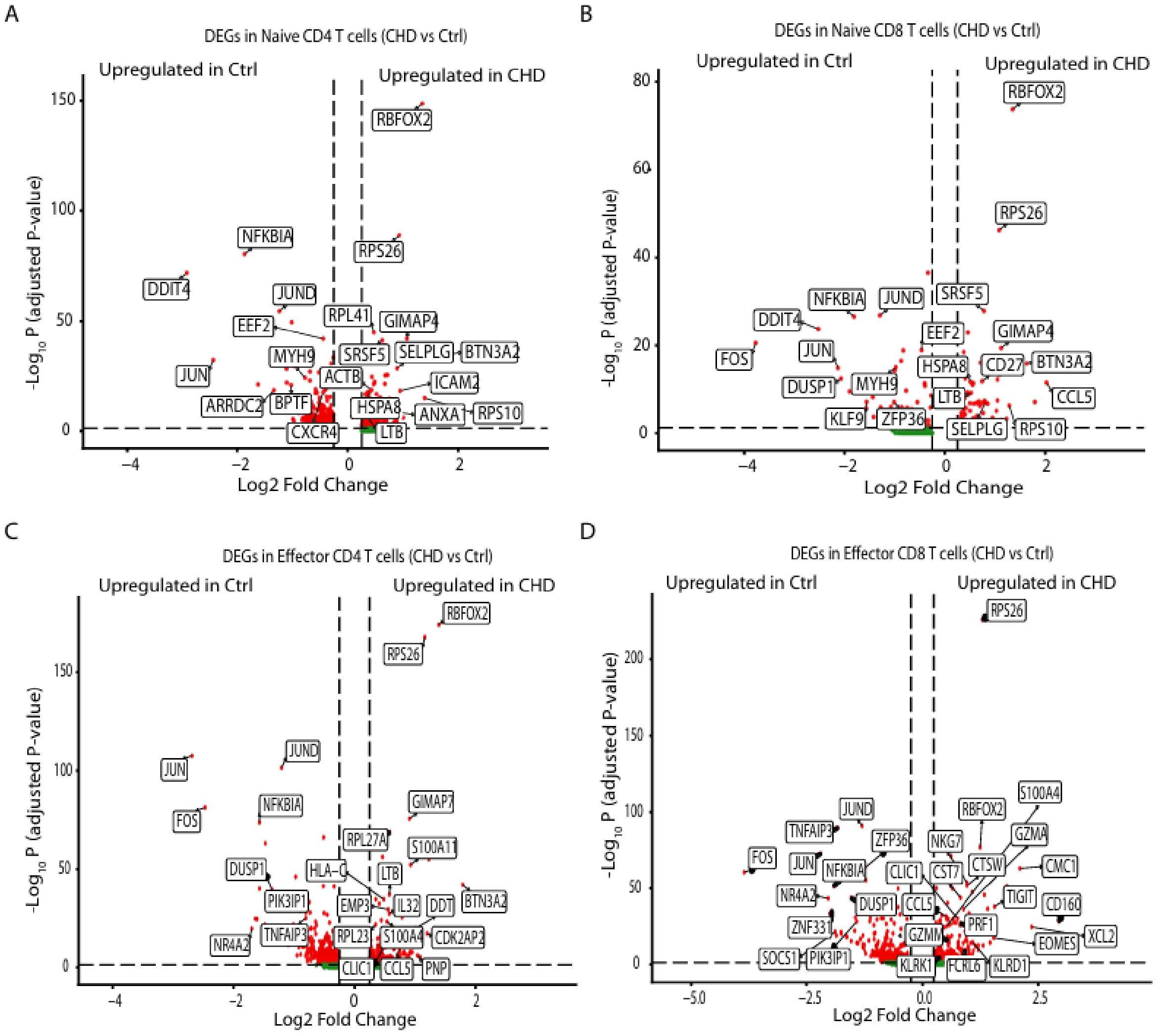

FIGURE S3: Increase in the expression of effector molecules in T cells of children with CHD. **A** - **D**) Volcano plot showing the differentially expressed genes in naïve CD4 T cells (**a**), naïve CD8 T cells (**B**), effector CD4 T cells (**C**) and effector CD8 T cells (GZMK^{hi} and GZMB^{hi} CD8 T) (**D**) between CHD and Ctrl. Each point represents a gene, with $\log_2$ fold change (x-axis) indicating the magnitude of expression change and $-\log_{10}$ adjusted p-value (y-axis) representing statistical significance. Red points indicate significantly significant genes (adjusted p-value < 0.05, $|\log_2 FC| > 0.25$), while green points represent non-significant genes. Selected top DEGs are labelled.

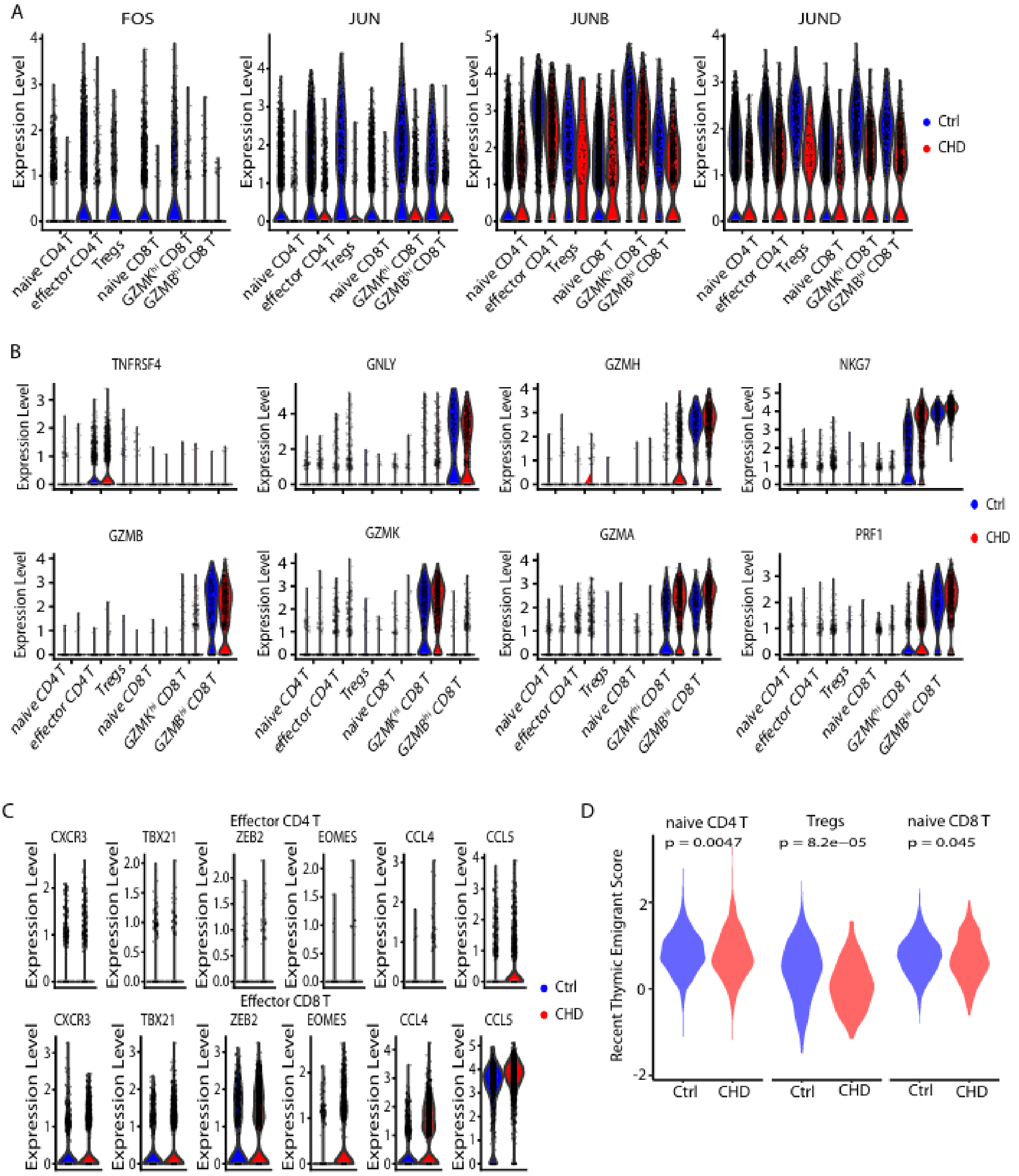

FIGURE S4: Down-regulated genes and Up-regulated genes per condition

A) Activator protein 1 (AP-1) transcription factor genes expressed per cluster in each condition as represented in the Violin plots **B**) Effector genes expressed per cluster in each condition as represented in the Violin plots. **C**) Senescent-associated genes and Type 1 Transcription factors genes expressed in effector CD4 and CD8 T cells per condition as represented in the Violin plots. **D**) Violin plot of the single-cell gene signature module score for recent thymic emigrant computed based on *PECAM1*, *SELL*, *CCR7*, *S1PR1*, *KLF2*, *LEF1*, *TCF7*, *SOX4*, *NT5E.*

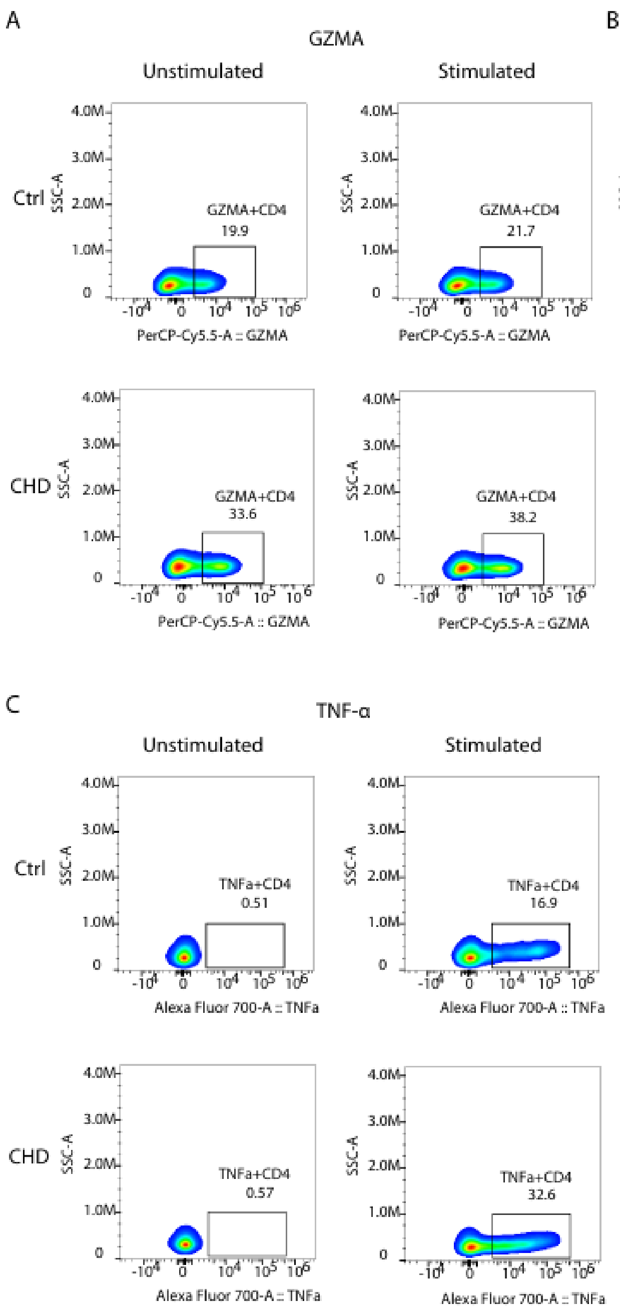

FIGURE S5: Intracellular Flow cytometry gating Strategy of Cytokines on CD4 T cells post-stimulation

A-C) Representative Cytometric gating strategy quantifying intracellular granzyme and cytokines from CD4 T cells after 3 hours PMA/Ionomycin Stimulation. Healthy Control (top panel) and CHD (bottom panel), unstimulated (left panel) and stimulated (right panel). **A**) GZMA. **B**) IFN-γ. **C**) TNF-α.

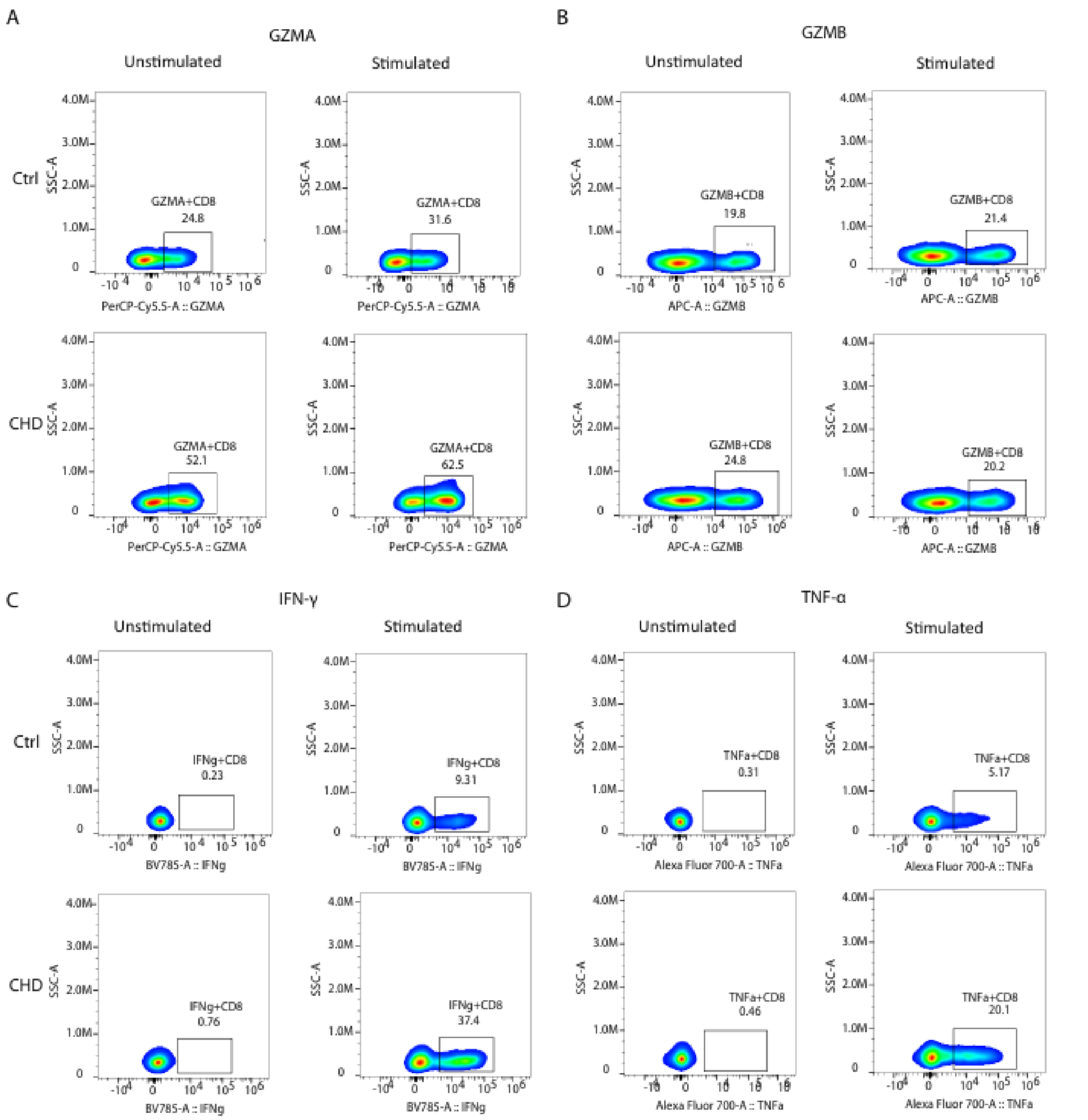

FIGURE S6: Intracellular Flow cytometry gating Strategy of Cytokines on CD8 T cells post-stimulation

A-D) Representative Cytometric gating strategy quantifying intracellular granzyme and cytokines from CD8 T cells after 3 hours PMA/Ionomycin Stimulation. Healthy Control (top panel) and CHD (bottom panel), unstimulated (left panel) and stimulated (right panel). **A**) GZMA. **B**) GZMB. **C**)IFN-γ. **D**) TNF-α.

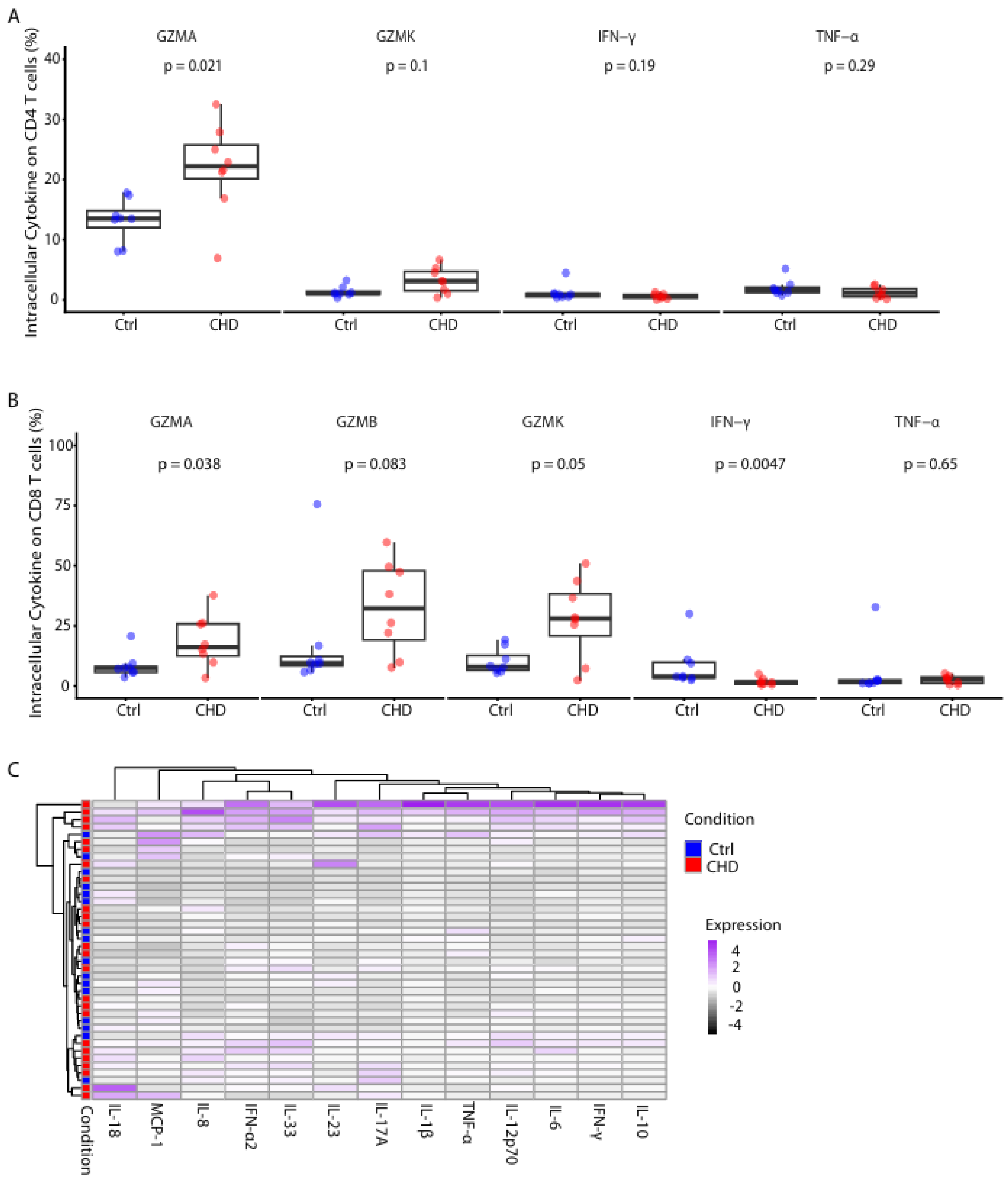

FIGURE S7: Higher granzyme producing T cells and Pro-inflammatory State in CHD

A) Percentage of intracellular cytokine detection of CD4 T cells among CHD and age-matched controls after 6 hours anti-CD3/anti-CD28 stimulation. **B**) Percentage of intracellular cytokine secretion by CD8 T cells among CHD and age-matched controls after 6 hours anti-CD3/anti-CD28 stimulation stimulation. **C**) Heatmap showing unsupervised clustering of Cytokine expression (columns) among the donor's group by Condition and Disease. Expression values were column-scaled to normalize across donors. The colour gradient (blue → white → red) represents low to high expression levels.

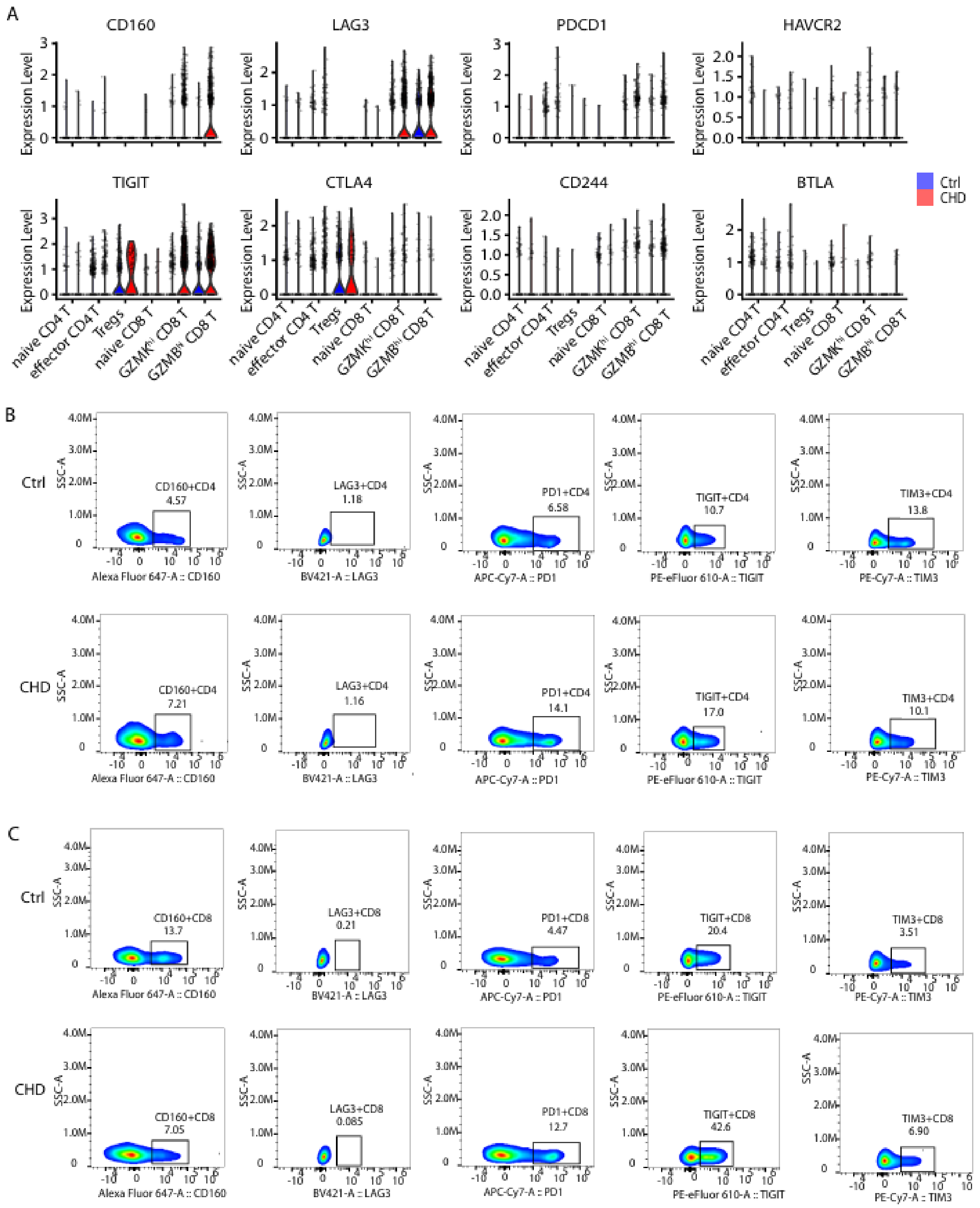

Figure S8: Higher Inhibitory receptors in T cells post-thymectomy in CHD Patients

A) Violin plots of Inhibitory receptors expression on the different cell clusters. **B**) Representative cytometric gating strategy quantifying inhibitory receptors expression on CD4 T cells in Healthy Control (top panel) and CHD (bottom panel) after 3 hours PMA/Ionomycin Stimulation. **C**) Representative cytometric gating strategy quantifying inhibitory receptors expression on CD8 T cells in Healthy Control (top panel) and CHD (Bottom panel) after 3 hours PMA/Ionomycin Stimulation

Table S1: Surface Antibodies Panel for Phenotyping

Fluorophores	Marker	Clone	Company	Dilution
BUV496	CD16	3G8	BD Horizon	1:100
BUV563	CD56	NCAM16.2	BD Horizon	1:100
BUV661	CD3	UCHT1	BD Horizon	1:100
BUV737	CD69	FN50	BD Horizon	1:100
BUV805	CD8a	SK1	BD Horizon	1:100
BV421	PD1	EH12.2H7	BioLegend	1:25
PB	CD57	HNK-1	BioLegend	1:100
VioGreen	Vd1	REA173	Miltenyi	1:200
BV570	HLA-DR	L243	BioLegend	1:100
BV605	CD45RA	HI100	BioLegend	1:200
BV650	CD127	A019D5	BioLegend	1:50
BV711	CCR6	G034E3	BioLegend	1:100
BV750	CD4	SK3	BioLegend	1:100
BV785	CCR7	G043H7	BioLegend	1:50
FITC	Vg9	REA470	Miltenyi	1:200
PerCP-Vio700	Vd2	REA771	Miltenyi	1:200
PE	TCRγδ	11F2	Miltenyi	1:200
PE-eFluor 610	NKG2A	REA110	Miltenyi	1:100
PE-Cy5	CD31	WM59	BioLegend	1:100
PE-Fire700	CD25	M-A251	BioLegend	1:100
PE-Cy7	CD19	HIB19	eBioScience	1:200
PE-Fire810	CCR4	L291H4	BioLegend	1:100
APC	CXCR3	1C6/CXCR3	BD	1:100
AF647	CD28	CD28.2	BioLegend	1:200
AF700	CD27	O323	BioLegend	1:400
Zombie NIR	Zombie NIR		BioLegend	1:800
APC-Cy7	CD161	APC-Cy7	HP-3G10	1:25

Table S2: Surface Antibodies Panel for Stimulation Assay

Fluorophores	Marker	Clone	Company	Dilution
BUV661	CD3	UCHT1	BD Horizon	1:100
BUV737	CD69	FN50	BD Horizon	1:100
BUV805	CD8a	SK1	BD Horizon	1:100
BV421	LAG3	11C3C65	Biolengend	1:100
BV570	HLA-DR	L243	BioLegend	1:100
BV750	CD4	SK3	BioLegend	1:100
FITC	Vg9	REA470	Miltenyi	1:200
PerCP-Vio700	Vd2	REA771	Miltenyi	1:200
PE	TCRgd	11F2	Miltenyi	1:200
PE-eFluor 610	TIGIT	MBSA43	Invitrogen	1:100
PE-Cy5	TCRab	11F2	Miltenyi	1:100
PE-Cy7	TIM3	F38-2E2	Biolengend	1:100
AF647	CD160	BY55	BioLegend	1:100
Zombie NIR	Zombie NIR		Biolengend	1:800
APC-Cy7	PD1	EH12.2H7	Biolegend	1:100

Table S3: Intracellular Antibodies Panel

Fluorophores	Marker	Clone	Company	Dilusion
BV785	IFNg	4S.B3	BioLegend	1:50
BV605	IL-4	MP4-25D2	BioLegend	1:100
PerCP-eFlour710	GZMK	G3H69	Miltenyi	1:50
PerCP-Cy5.5	GZMA	CB9	Biolegend	1:50
APC	GZMB	GB11	BioLegend	1:50
AF700	TNFa	MAb11	BioLegend	1:50

Table S4: Cytokine Essay relating to Figure 4 Plasma cytokine Detection levels

Cytokines	LOD (pg/mL)
IL-1β	5.12
IFN-α2	2.4
IFN-γ	6.202
TNF-α	17.22
MCP-1	16.93
IL-6	7.227
IL-8	65.37
IL-10	4.44
IL-12p70	3.76
IL-17A	1.035
IL-18	10.13
IL-23	12.79
IL-33	5
